# Let’s Get Cooking: a mixed methods evaluation of a programme delivering culinary education via a network of cookery clubs across England

**DOI:** 10.1017/S1368980026102675

**Published:** 2026-05-21

**Authors:** Georgia Browne, Rachel Gibson, Suzanne Mitchell, Fiona Lavelle

**Affiliations:** 1 Department of Nutritional Sciences, King’s College London, UK; 2 British Dietetic Association, UK

**Keywords:** Cooking, Sustainability, Train the trainer, Food waste, Behaviour change techniques

## Abstract

**Objective:**

Culinary education interventions may effectively improve cooking skills and dietary outcomes; however, interventions lack theoretical underpinning and rigorous evaluation. ‘Let’s Get Cooking’ (LGC) is a culinary education intervention operating a ‘school and community-based’ programme targeting children and ‘food waste reduction’ programme targeting adults. LGC is delivered through cookery clubs, operating a ‘train the trainer’ model, whereby club leaders are centrally trained and resourced. This study aims to understand initial assessments of LGC’s effectiveness, the content of resources provided for programme delivery and club leaders’ perceptions of the programme.

**Design:**

Three previous evaluations of the ‘school and community-based’ programme were synthesised and critically appraised. LGC resources were coded to identify behaviour change techniques (BCT), map the age-appropriateness of included cooking skills and to identify food waste reduction strategies. Qualitative one-on-one interviews with club leaders (*n* 7) were conducted online and thematically analysed.

**Setting:**

UK

**Participants:**

N/A

**Results:**

Previous evaluations suggest LGC’s ‘school and community-based’ programme may effectively disseminate cooking skills. However, reports lacked assessment of ‘fidelity’ or ‘dose delivered’. LGC resources primarily target behaviour change through describing how and when to perform cooking behaviour and contain age-appropriate cooking skills for participating children. ‘Food waste reduction’ resources primarily include appropriate food storage and preparation strategies to reduce waste. Club leaders perceive the intervention and training positively, however, adapt resources and sessions due to delivery challenges.

**Conclusions:**

LGC may be an effective intervention; however, content improvement may support programme delivery. Rigorous process evaluation is needed to improve understanding of LGC’s effectiveness.

Poor diet quality is implicated in the rapid global increase in non-communicable diseases, posing detrimental risk to long-term physical and mental health^(1)^. Cooking skills (physical or mechanical skills used in the production of a meal, e.g. peeling and boiling^(2)^) and food skills (knowledge and skills to be able to select and prepare food, e.g. shopping and meal planning^(2)^) have been associated with better diet quality^(3)^, reducing convenience food consumption^(4)^ and increasing self-preparation^(5)^. Food skills may additionally facilitate intention to reduce household food waste^(6)^, minimising its associated negative socioeconomic and environmental impacts, which are argued to be largely avoidable^(7)^.

Early acquisition of cooking skills may confer long-term beneficial effects on cooking behaviours, diet quality and food waste reduction into adulthood^(8)^. However, evidence suggests transference of cooking skills from parents to children is declining^(9)^. Time scarcity, both real and perceived, is a barrier to children’s involvement in food preparation^(9)^, as are concerns that children lack the necessary fine motor skills to prepare food safely^(9)^. Additionally, a general population-level decline in adequate cooking skills for food preparation^(10)^ may further inhibit social modelling. Given the sociodemographic variation in cooking skills and attitudes^(5,11)^, particularly the association between lower educational attainment and lower cooking and food skills confidence^(3)^, this may disproportionately affect children in areas of deprivation.

Consequently, the importance of prevention strategies which target health and nutrition literacy to improve diet-related outcomes is recognised^(12)^. A wide range of culinary education interventions have been developed to target children, adults and families, varying in setting, approach, duration and dose^(13–16)^. Overall, interventions have demonstrated improvements in nutrition-related behaviours (e.g. fruit and vegetable consumption), attitudes (e.g. willingness to try new foods) and psychosocial outcomes (e.g. cooking self-efficacy)^(14–16)^. Recent frameworks for developing culinary education programmes also integrate components of food waste education^(17),^ and evidence suggests targeting food management skills (e.g. storage, meal planning) may be effective for minimising household food waste^(18)^.

However, culinary education interventions have been criticised for weaknesses in methodological design^(13,15,16)^, a lack of theoretical underpinning and opacity about how any applied theories inform programme design^(13,14,16,19)^. For example, programmes targeting children often lack due consideration of the age-appropriateness of their content^(16,17)^. Heterogeneity in study design has also limited the identification of which programme characteristics contribute to observed effects^(13,15,16)^. Furthermore, specific techniques for changing target behaviours are rarely reported^(19)^, for example referencing established behaviour change technique (BCT) taxonomies^(20,21)^. This prevents the characterisation of the active components of an intervention and limits the understanding of ‘what works’ in different populations and contexts^(22)^. Validated measures for influences on cooking behaviours, such as cooking skills, food skills, and perceived cooking confidence, have been developed to act as a proxy for cooking (which lacks validated measures)^(23)^; however, these are infrequently utilised^(13,15)^. A lack of process evaluation has limited the production of evidence to justify ongoing support for interventions and investigate their scalability, replicability and areas for refinement^(23)^. Thorough evaluation is critical to investigate contextual factors impacting delivery and observed outcomes^(24)^. Only recently has a specific framework for systematically developing and evaluating culinary interventions been developed, namely the ‘Cook-Ed^TM^’ model^(17,23)^, which sets out an eight-stage, end-to-end co-design process for culinary education programmes aimed at improving skills for healthy food preparation. The Cook-Ed^TM^ model consists of three phases: ‘planning’ (stages 1–3), which defines the cooking-related need, considers target behaviour change factors in programme design and assesses capacity for delivery; ‘development and implementation’ (stages 4–5), which develops and tests programme content, defines evaluation measures, and assesses programme feasibility; and, finally, ‘evaluation’ of process, impact and outcome measures using validated tools (stages 6–8)^(23)^. Consultative engagement with key stakeholders is encouraged throughout^(23)^. Cook-Ed^TM^ components may also be pragmatically applied to existing programmes to inform their iterative refinement.

‘Let’s Get Cooking’ (LGC) is a culinary education intervention, which was established by the Children’s Food Trust (formerly the School Food Trust) in 2007. LGC was designed to be delivered through a cookery club format, operating a ‘train the trainer’ model whereby LGC club leaders are centrally trained and provided resources and funding to establish clubs in their various contexts. LGC initially established a ‘school and community-based’ programme for children and their families in England, prioritising the most disadvantaged areas. By 2012, the ‘school and community-based’ programme was established nationally with c.5000 cookery clubs, c.3000 of which were newly established and c.2000 of which were existing clubs integrated into the LGC network^(25)^. By September 2017, over three million people had benefitted from the ‘school and community-based’ programme^(26)^, at which time ownership of LGC transferred to the British Dietetic Association (BDA) following the closure of the Children’s Food Trust. Under the BDA’s leadership, LGC’s ‘school and community-based’ programme continues, with the aim of equipping children and families with cooking and food skills to prepare healthy food and improve dietary intake; however, the number of clubs and programme reach has been in decline. In 2015, an additional LGC programme for adults focusing on ‘food waste reduction’ was established in partnership with the Merseyside Recycling and Waste Authority. Several public evaluations of LGC’s ‘school and community-based’ programme were published under the Children’s Food Trust with varying scopes and sub-population focuses^(25–28)^. However, the last of these evaluations was conducted in 2017, since which time the ‘school and community-based’ programme has evolved. No formal evaluation of LGC’s ‘food waste reduction’ programme has been conducted to date. Furthermore, the design of the LGC intervention was not underpinned by specific theoretical frameworks; thus, the active components which characterise this intervention are currently unknown.

As outlined by the Cook-Ed^TM^ model, understanding the current content and delivery of LGC and the findings and robustness of previous evaluations is essential to facilitate programme refinement^(23)^. Therefore, the aim of this study is to understand initial assessments of LGC’s effectiveness, the content of resources provided for programme delivery, and the experiences and perceptions of LGC club leaders. The objectives of this study are as follows: (1) to synthesise and critically appraise previously published evaluations of LGC’s ‘school and community-based programme’; (2) deconstruct LGC programme resources and assess their alignment to theoretical frameworks; and (3) conduct interviews to understand LGC club leaders’ perspectives on the programme and training. These reflect a pragmatic application of principles outlined in the ‘planning’ stages of the Cook-Ed^TM^ model to inform the refinement of LGC’s programme and evaluation design. This includes reviewing existing evaluations to identify evidence gaps and methodological limitations (Objective 1, Cook-Ed^TM^ stage 1): understanding the content of programme resources to identify theoretical and practical factors unaddressed by previous evaluations (Objective 2, Cook-Ed^TM^ stage 2) and consulting key stakeholders to assess delivery capacity, triangulate and contextualise findings (Objective 3, Cook-Ed^TM^ stage 3)^(23)^.

## Methods

The three components of this mixed methods study have been specifically chosen to understand the existing evidence base, current content and delivery of LGC, respectively, to inform its future refinement, reflecting the ‘planning’ principles described in Cook-Ed^TM^ model stages 1–3^(23)^. The three components include the following: (1) synthesis and critical appraisal of previous LGC evaluations (Objective 1, Cook-Ed^TM^ stage 1), (2) deconstruction of LGC resources (Objective 2, Cook-Ed^TM^ stage 2) and (3) club leaders’ perspectives on LGC (Objective 3, Cook-Ed^TM^ stage 3).

### Synthesis and critical appraisal of previous LGC evaluations

An online search of material in the public domain identified a total of three previous evaluations of LGC’s ‘school and community-based’ programme with publicly available reports. This included a five-year ‘Evaluation of the Let’s Get Cooking Programme’ conducted from the programme’s inception in 2007–2012^(25,28)^, a 2016 evaluation of the programme focused on ‘looked after children’^(27)^ and a two-year evaluation of ‘school-based clubs’ conducted from 2015 to 2017^(26)^. No publicly available evaluations of LGC’s ‘food waste reduction’ programme were identified.

Evaluation reports were read several times for full immersion. A data extraction tool was developed to facilitate the relevant information extraction. Quantitative and qualitative data on the following topics were extracted from each evaluation for synthesis: programme aims and design, evaluation methodologies, facilitator feedback, programme cost and participant outcomes. Participant outcomes were summarised by programme and are presented in Table 1; a specific theoretical framework was not applied. Extracted data on evaluation methodologies was systematically compared to the elements of Saunders *et al.*’s process evaluation framework^(24)^: fidelity, dose delivered, dose received, reach, recruitment and context. A table of included and excluded components in each evaluation was compiled. Due to variation in reported detail, data from the most comprehensive report (five-year ‘Evaluation of the Let’s Get Cooking Programme’ 2012^(25,28)^) were synthesised first, and data from the remaining reports in ‘school-based clubs’^(26)^ and ‘looked after children’^(27)^ were compared for pertinent similarities and differences. Results were summarised descriptively (Section 3.1). Full details of evaluation methodologies, programme aims, and design are given in online Supplementary Material A Tables A.1/A.2.

**Table 1.** Assessment tools and synthesis of reported outcomes in previously published Let’s Get Cooking evaluations Table 1 long description.

Evaluation	Assessment tools	Validated assessment tools	Participant outcomes				
			Reach	Learning and replicating skills	Improving eating habits	Passing knowledge to others	Psychosocial benefits
Five-year ‘Evaluation of the Let’s Get Cooking Programme: Final Report’ and Summary, 2012^(25,28)^	Self-report participant questionnaire at baseline and 3-month follow-up (children key stage 2 and over) OR self-complete picture card exercise (children key stage 1 and under); coded qualitative feedback from club leaders, participants and caregivers; and routine online activity reports by club leaders.	Not reported.	Total reach of c.1·8 million people^^*^^ (574 318 club members, 700 667 home beneficiaries^^*^^ and 498 985 community members). Club members were on average 9·7 years of age and 39 % male. A network of over 5000 clubs was established across all local authorities in England.	92 % of participants replicated at least one skill at home during Let’s Get Cooking and 91 % in the 3-months following. ‘Follow a recipe’ and ‘cut, chop, slice food’ were the most common skills learned and replicated. Safety skills more frequently recalled by participants compared to cooking skills at 3-month follow-up.	58 % of participants reported at least minimal improvement in consumption of ‘healthy’ items^†^; of these 16 % reported significant improvement. 45 % of children maintained any improvement in dietary intake at 3-month follow-up compared to baseline^†^. No improvement in mean number of healthy food items consumed by infants was observed. No significant differences in dietary outcomes by gender or club series length. Uptake in school lunches increased short-term but declined at 3-month follow-up, subject to significant seasonal confounding.	81 % of participants reported family and friends watching or helping them replicate skills at home. On average, participants passed knowledge onto 1·22 individuals^^*^^.	Enjoyment, teamwork, fun, opportunities to socialise and meeting new people.
‘Cooking with Looked After Children: Evaluation Report’, 2016^(27)^	Participant cooking journals and focus groups with carers.	Not reported.	Trained 137 foster carers and residential children home staff.	Carer feedback suggests cooking, food hygiene and safety skills disseminated. 35 of 37 possible recipes provided were replicated. 92 % of participants agree they would cook recipes again.	86 % of children tried a new food and vegetables represented 13 % of new foods tried.	Not reported.	Relationship development and improved confidence, communication and social skills.
‘Let’s Get Cooking: School-based Clubs (2015–2017) Report Summary’^(26)^	Routine online feedback forms and annual surveys by club leaders. Qualitative interviews with club leaders.	Not reported.	Over 158 657 children and 23 866 adults from 2015–2017.	Improved cooking skills (e.g. safe techniques for cutting) and food hygiene awareness.	Trying new foods.	Not reported.	Improved confidence, self-esteem or self-belief, enjoyment, interest and opportunities for bonding and family socialisation

* The number of home beneficiaries was estimated using the assumption that each club member passed skills onto an additional 1·22 people on average.

† Improvement defined according to a healthy score increase: ≥ 1 point = minimal; ≥ 10 points = significant; healthy score = number of healthy items – number of unhealthy items, as indicated through a self-complete questionnaire (see online Supplementary Material A Table A.1).

### Deconstruction of LGC resources

Resources used in the training and delivery of LGC’s ‘school and community-based’ and ‘food waste reduction’ programmes were provided by the BDA. Due to the volume of resources used across the duration of the LGC programmes and project time constraints, a sample deemed representative of the overall suite of resources was selected by the LGC programme lead at the BDA. Resources were categorised by type including ‘training’ resources for BDA staff, ‘logistics’ resources for club leaders (e.g. safety checklists), resources for ‘session delivery’ and ‘educational’ material distributed to participants. Several ‘session delivery’ and ‘educational’ resources also included printed recipes.

Three deconstruction processes were conducted: coding of BCT, assessment of age-appropriateness (‘school and community-based’ programme resources only) and identification of food waste reduction strategies (‘food waste reduction’ programme resources only). Full details of deconstructed resources are provided in Table 2. A second researcher independently coded 11 % of resources, which was deemed a sufficient proportion to assess inter-rater reliability. Overall, inter-rater relatability for included codes and frequency of occurrence of codes was 95 % and 77 %, respectively, as measured by percentage agreement (details in online Supplementary Material B). Coding discrepancies were discussed, and agreement reached.

**Table 2.** Deconstruction processes conducted by resource Table 2 long description.

Programme	Resource type	Resource name	Description	BCT mapping	Age-appropriateness assessment	Food waste reduction strategy identification
School and community-based	Session delivery incl. recipes	Favourite takeaways to cook at home	Termly activity pack containing take-away inspired activity ideas for programme facilitators and 17 take home recipes for participants	✓	✓	
		Fruit & berry recipes and activity ideas	Termly activity pack containing fruit-related activity ideas for programme facilitators and 12 take home recipes for participants	✓	✓	
		Ideas and recipes for getting back to basics	Termly activity pack containing ‘back to basics’ activity ideas for programme facilitators and 12 take home recipes for participants	✓	✓	
	Session delivery	Dissect your pie	Interactive activity to learn about food hygiene	✓		
		Cooking and demonstrating with groups	Preparation and delivery tips for club leaders to effectively cook with groups	✓		
		Guide to running cooking sessions	Guidance for club leaders on running cooking sessions including programme planning, recruitment, session organisation and content	✓		
		Before You start cooking	Informational poster with six steps for food hygiene and safety before beginning food preparation	✓		
	Educational	A Guide to using LGC recipes	A guide on how to use LGC recipes and an explanation of the different kinds of information included	✓		
		Hygiene and safety checklist	Checklist of food hygiene and safety items to be used by participants at home	✓		
		Perfect portions	Guidance for participants on appropriate portion sizes of different food groups for both children and adults	✓		
Food waste reduction	Session delivery	Before you start cooking	Informational poster with six steps for food hygiene and safety before beginning food preparation	✓		
		Dissect your pie	Interactive activity to learn about food hygiene	✓		
		A Guide to running cooking sessions	Guidance for club leaders on running cooking sessions including programme planning, recruitment, session organisation and content	✓		✓
	Educational incl. recipes	Use your loaf	Information on household waste of bread and recipes and suggestions to minimise it	✓		✓
		Make the most of your mince	Information on economical and health benefits of mince, with recipes and suggestions on how to prepare it	✓		✓
		Soup up your leftover vegetables	Information on household waste of vegetables and recipes and suggestions to minimise it	✓		✓
	Educational	Freezing, batching, storing	Educational sheet with ideas to save money and prevent food waste	✓		✓
		Go potty with potatoes	Information on economic and health benefits of potatoes, with suggestions on how to store and links to online recipe resources	✓		✓
		Cooking skills	Visual representation of commonly used cooking skills in LGC sessions	✓		
		Perfect portions	Guidance for participants on appropriate portion sizes of different food groups for both children and adults	✓		

BCT, behaviour change techniques; LGC, Let’s Get Cooking.

#### BCT coding

LGC does not have a theoretical underpinning and LGC resources did not explicitly report BCT. Consequently, a deductive coding approach was applied to identify BCT using Michie *et al.*’s ‘Coventry, Aberdeen & London – Refined’ (CALO-RE) taxonomy^(21)^ (see online Supplementary Material C Table C.1 for a complete list of BCT definitions). BCT were coded where identifiable targeting cooking as a behaviour. The total occurrence and percentage of resources each BCT occurred in was analysed by programme. Examples of how identified BCT targeted cooking as a behaviour were recorded to provide evidence of their application in LGC resources. The CALO-RE taxonomy was selected over alternative taxonomies, such as the Behaviour Change Technique Taxonomy v1^(20)^, for its specific relevance to interventions targeting behaviours to improve diet quality^(21,23)^ and to enable direct comparison to literature^(19)^.

#### Age-appropriateness assessment

A random sample (51 %, *n* 21) of recipes included in ‘session delivery’ resources was selected for deconstruction using a random number generator. The distribution of selected recipes from relevant ‘session delivery’ resources and different cuisines/recipe types was checked to ensure a representative sample. Recipes were deductively coded using Dean *et al.*’s guidelines^(29)^ to identify all included cooking skills and their corresponding minimum age requirements. Only cooking skills in the ‘method’ section of each recipe were coded as these were deemed essential to its preparation. Where an individual recipe contained variations of a single cooking skill requiring significantly different techniques, these were coded as separate occurrences, for example, using ‘weighing scales’ and ‘measuring spoons’ or ‘finely slicing’ and ‘chopping’ represents two occurrences of ‘weighing and measuring’ and ‘cutting, chopping and slicing’ respectively. A subjective assessment of additional factors affecting a recipe’s age-appropriateness, such as use of appropriate language, font size and clear page layouts, was also assessed in line with elements discussed by Dean *et al.* ^(29)^.

#### Food waste reduction strategy identification

‘Food waste reduction’ programme resources were inductively coded to create a comprehensive codebook of food waste reduction strategies (e.g. repurposing leftovers), which were checked for overlap and grouped according to the ‘household food-related practices and lifestyle’ categories as described by Chia *et al.* ^(30)^ (e.g. cooking and leftover reuse; etc.) to facilitate comparison to literature. Resources were then re-read and the frequency of occurrence of each identified food waste reduction strategy in the codebook recorded.

### Club leaders’ perspectives on LGC

Researchers adopted a realist ontology^(31)^ and essentialist epistemology. A qualitative descriptive design was conducted using a pragmatic approach to inform strengths and recommendations relating to the LGC programme and training, with themes identified at the semantic level^(32)^.

Convenience sampling was used. Recruitment advertisements were shared by the BDA via email; all subsequent contact proceeded directly with participants via email and the BDA had no knowledge of participating individuals. Eligible participants were LGC club leaders who had completed training and run a programme at any time since 2017. Exclusion criteria included being under 18 years old. Participants were provided an information sheet and gave written consent. Participants were aware they were not obliged to participate and could withdraw up to the date of report submission for final review without reason or consequence. No relationship was established with participants prior to study commencement nor was personal researcher characteristics disclosed. Participants were aware of the study aims as related to interviews. Interviews were conducted by a female researcher (GB), with an MChem qualification, completing an MSc in Nutrition and 2–3 years’ experience interviewing subject experts in corporate consultancy. Interviews were conducted one-on-one, online via Microsoft Teams in July 2024. Interviews lasted between 23–44 min. No repeat interviews were conducted. A semi-structured format with guided open-ended questions was used (see online Supplementary Material D for interview schedule). No pilot testing was conducted due to time constraints. Participants received an honorarium £25 voucher for their time. See online Supplementary Material E Table E.1 for a completed ‘Consolidated Criteria for Reporting Qualitative Research’ (COREQ) checklist.

Interviews were audio recorded, transcribed verbatim and checked for accuracy against field notes. Participants weren’t contacted for comment on transcripts or study findings. Data were coded by a single researcher, and an inductive thematic analysis was conducted in line with Braun and Clarke^(32)^ using NVivo 14 software (QSR International Pty Ltd, Australia). Data within codes were checked for coherence and codes grouped were into themes. Themes were checked for overlap to ensure ‘clear and identifiable distinctions’^(32)^. Illustrative quotes were extracted from data where appropriate. A collection of additional extracts mapped to each theme can be found in online Supplementary Material F.

## Results

### Synthesis and critical appraisal of previous LGC evaluations

Previous evaluations of LGC’s ‘school and community-based’ programme suggest it effectively disseminated cooking skills and modestly increased healthy food consumption (Table 1). Facilitator feedback collected via post-training feedback forms suggest training was highly rated by club leaders, with between 96–100 % rating training as ‘excellent’ or ‘very good’ across evaluations.

The extent to which methodological detail was provided varied considerably between evaluation reports, limiting comparability. The initial five-year evaluation final report provided information pertaining to study design, for example sampling, method, questionnaire development and testing, fieldwork, data collection and statistical analysis. In contrast, the evaluation reports of ‘school-based clubs’ and ‘looked after children’ provided less methodological information. Although sample sizes were given in the ‘school-based clubs’ report, information on sampling methodologies was lacking; for example, the sampling methodology of club leaders to participate in qualitative interviews was not provided. Additionally, it is unclear exactly what types of data were collected and analysed to produce presented results, such as how data from cooking journals in ‘looked after children’ were used to determine the proportion of new foods tried which were vegetables.

It is unclear whether methods of participant outcome measurement were validated in studied populations across reports. The initial five-year evaluation of LGC describes testing the self-report questionnaire for participants in a sample of clubs to determine ease of use and, however, does not provide information on the validity of this method in the target population. Within the ‘school-based clubs’ report, club leader feedback was used to assess the impact of LGC on participants and the validity of this method to assess presented outcomes such as ‘improving literacy and numeracy skills’ is unknown.

Regarding process evaluation, only the initial five-year evaluation of LGC analysed the impact of contextual factors such as gender, club series length and seasonality (e.g. variable school meal uptake) on observed outcomes and acknowledged that participating schools may be relatively more engaged with the school food agenda. However, the impact of contextual and intervention-specific differences between new and associate clubs (e.g. differing funding and training structures) on programme outcomes and implementation was not presented.

No evaluation explicitly defined acceptable programme delivery in line with an underpinning theory or presented an assessment of programme ‘fidelity’^(24)^. Although the ‘school-based clubs’ and ‘looked after children’ evaluations provided details of training content and delivery, no underlying theoretical assumption was referenced.

In terms of ‘dose delivered’, the five-year evaluation described series length as varying ‘considerably’ and however did not outline the intended number of sessions per series or a timeframe for their delivery. Evaluations in ‘school-based clubs’ and ‘looked after children’ did specify a ‘dose’; however, adherence was not reported. No evaluation reported the extent to which content was covered or resources used as intended within sessions. Furthermore, the impact of socioeconomic, environmental or resource-related factors on programme implementation was not commonly discussed and only the ‘school-based clubs’ evaluation reported feedback on challenges in programme delivery, namely lack of funding, facilities, cooking equipment and time. Only the five-year evaluation provided information on programme cost, which was £34 per club member and £11 per beneficiary in 2012.

All evaluations included some assessment of the ‘dose received’ by participants and defined expected engagement behaviours. However, only the five-year and ‘looked after children’ evaluations directly measured these. All evaluations measured overall programme’s ‘reach’. However, recruitment methodologies, proportional reach in priority populations (e.g. schools with high free school meal eligibility) and barriers to participation were not reported.

### Deconstruction of LGC resources

Twenty resources were identified as appropriate to be coded for BCT, three for age-appropriateness assessment and six for food waste reduction strategy identification (Table 2).

#### BCT Coding

Overall, 18 of 40 BCT in the CALO-RE taxonomy^(21)^ were identified across resources (Table 3), with between 1 and 14 BCT present per resource. Most unidentified BCT (e.g. BCT#31–40) were not applicable to practical interventions, however others were (e.g. BCT#9 *set graded tasks*).

**Table 3.** Total occurrence by BCT and programme Table 3 long description.

BCT (as defined by CALO-RE taxonomy (Michie *et al.*, 21))	Example of BCT targeting cooking behaviour	Total occurrence by LGC programme (% of total programme resources)			
		School and community-based		Food waste reduction	
		*n*	%	*n*	%
21. Provide instruction on how to perform the behaviour	Distribution of resources with step-by-step recipes and explanations of how to carry out proper food hygiene and safety practices	55	90	55	100
20. Provide information on where and when to perform the behaviour	Instructing participants on food hygiene behaviours to be carried out before food preparation	23	70	29	60
2. Provide information on consequences of behaviour to the individual	Discussions of the benefits of food safety practices, such as lower risk of personal injury	11	60	23	70
26. Prompt practice	During sessions, participants are involved in practical preparation of recipes and engaged in food hygiene and safety behaviours	25	70	6	40
1. Provide information on consequences of the behaviour in general	Information on how certain cooking skills and techniques can alter the vitamin content or freshness of food	8	40	10	60
22. Model/Demonstrate the behaviour	Practical sessions include full or partial demonstration of recipe preparation by programme facilitators	10	60	4	20
15. Prompting generalisation of a target behaviour	Prompting participant discussion of possible ingredient substitutions within recipes for replication	5	50	2	20
4. Provide normative information about others’ behaviour	Contextualising difficulties the general population faces with cooking skills, for example the identification of portion sizes	2	20	4	40
5. Goal setting (behaviour)	Encouraging participants to identify cooking skills they would like to learn during practical sessions	3	10	2	10
8. Barrier identification/problem solving	Participants asked to identify how food hygiene risks could be prevented in the future	2	20	3	30
18. Prompting focus on past success	Programme facilitators encouraged to praise participants for cooking skills acquired in previous sessions	4	40	1	10
10. Prompt review of behavioural goals	Prompting participant reflection and discussion of cooking skills learned at the end of practical cooking sessions	2	10	3	10
12. Prompt rewards contingent on effort or progress towards behaviour	Distribution of prizes for learning information about the nutritional composition of foods (as opposed to rewards for directly improving diet quality)	2	10	2	10
13. Provide rewards contingent on successful behaviour	Distribution of prizes for learning new cooking skills during practical cooking sessions	1	10	1	10
29. Plan social support/social change	Designating the role of facilitators to assist participants with more difficult tasks during practical session	1	10	1	10
30. Prompt identification as role model/position advocate	Participants encouraged to demonstrate and share their cooking skills with others, for example at community events	1	10	1	10
24. Environmental restructuring	Acquiring store cupboard ingredients to enable the preparation of quick meals under time pressure, rather than opting for take-away foods	0	0	1	10
7. Action planning	Planning portion sizes depending on the size and hunger of the group being fed	0	0	1	10

LGC, Let’s Get Cooking; BCT, behaviour change techniques.

The ‘school and community-based’ and ‘food waste reduction’ programmes shared four of their five most frequently occurring BCT, namely BCT#21 *provide instructions on how to perform the behaviour*, BCT#20 *provide instruction on where and when to perform the behaviour*, BCT#2 *provide information on consequences of the behaviour to the individual* and BCT#26 *prompt practice*. Instructions on how (BCT#21) and when (BCT#20) to perform cooking behaviours occurred in 95 % and 65 % of resources, respectively. Across both programmes, information on the individual consequences of cooking behaviours (BCT#2) primarily referenced self-preparation being ‘quicker, cheaper and healthier’ than convenience alternatives. Resources frequently prompted practice (BCT#26), reflecting LGC’s ‘hands-on’ approach and encouragement of behaviour replication at home. ‘School and community-based’ resources frequently included facilitator demonstration (BCT#22 *model/demonstrate the behaviour*). Over half of ‘food waste reduction’ resources referenced the negative environmental impacts of food waste (BCT#1 *provide information on the consequences of the behaviour in general)*.

#### Age-appropriateness Assessment

**Figure 1.**
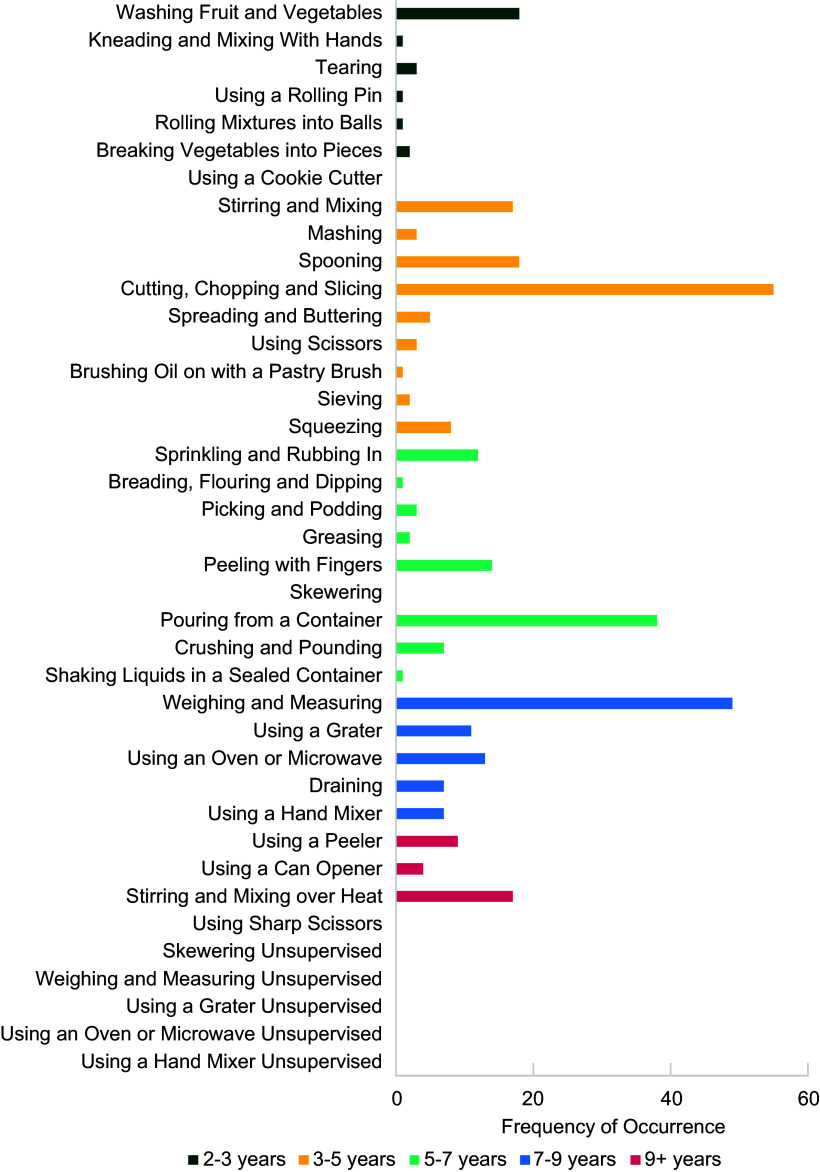
Figure 1 long description. Total occurrence in deconstructed recipes by cooking skill.

Individual recipes included between 7–17 separate cooking skills. As illustrated by Figure 1, the most commonly occurring cooking skills were ‘cutting, chopping and slicing’ (3–5 years), ‘weighing and measuring ingredients’ (7–9 years) and ‘pouring from a container’ (5–7 years).

Cooking skills appropriate for 3–5-year-olds occurred most frequently, representing 34 % of identified skills, followed by 7–9 years (26 %), 5–7 years (23 %), 9+ years (9 %) and 2–3 years (8 %). No recipe specified an appropriate age-range and at most, 50 % of cooking skills in each recipe originated within a single age group. About one-fifth (19 %) of recipes did not contain cooking skills suitable for the youngest (2–3 years) or oldest (9+ years) age groups.

Layout was consistent across recipes, outlining ingredients, equipment and method. However, some included sections relating to food resource management, such as how to ‘prepare now, eat later’, may not be appropriate for children. Language used within methods was in some cases complex or lacked step-by-step instructions for performing certain cooking skills (e.g. deseeding a chilli) or when and how to use specific equipment (e.g. measuring spoons *v*. measuring jugs). Furthermore, font was small, and methods in deconstructed recipes were not accompanied by visual aids such as pictures.

#### Food Waste Reduction Strategy Identification

**Figure 2.**
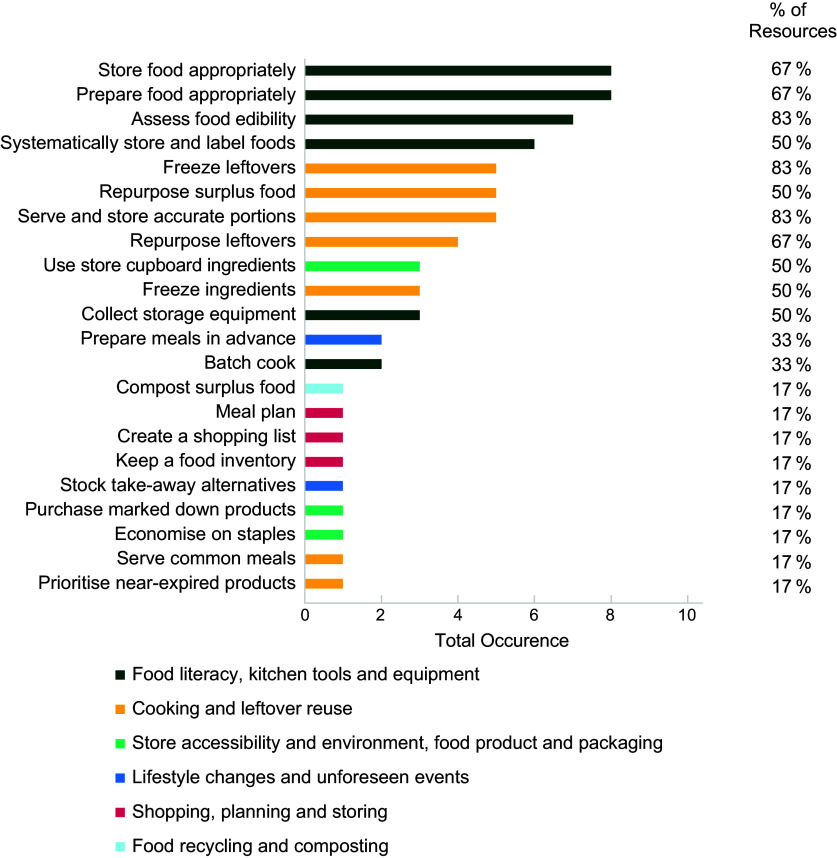
Figure 2 long description. Total and percentage occurrence in deconstructed resources by food waste prevention strategy.

The most frequent food waste reduction strategies identified related to ‘food literacy, kitchen tools and equipment’ and ‘cooking and leftover reuse’ (Figure 2), occurring at least twice per resource. Strategies encompassed the food preparation and storage life cycle; most resources (67–83 %) included at least one strategy for assessing edibility (e.g. visual assessment or best before dates) or preparing and storing food appropriately to prolong shelf life, facilitated, for example, by keeping empty ‘plastic tubs’ for storing leftovers. Most resources (67–83 %) contained information on repurposing leftovers or surplus foods, for example by preparing mince from leftover cooked meat. Half of resources discussed serving and storing accurate portions when preparing or freezing food. One resource discussed prioritising the use of near-expired products.

Strategies within ‘store accessibility and environment, food product and packaging’ primarily discussed using long-life store cupboard ingredients to ‘bulk out’ recipes which use surplus foods. Only 10 % of strategies identified related to ‘lifestyle changes and unforeseen events’, ‘shopping, planning and storing’ or ‘food recycling and composting’.

### Club leaders’ perspectives on LGC

Twelve individuals responded to the invitation to participate in interviews, nine consented and eight interviewed, with one withdrawal due to illness. Seven interviews were analysed, with one participant excluded due to answers indicating that they didn’t lead their club, despite confirming their participation in training. Participant demographics are shown in Table 4.

**Table 4.** Participant characteristics Table 4 long description.

LGC Programme	Code	Gender	Age (years)	Region	Club maturity (years)	Club setting	Club target age group
School and community-based	LGC001	Woman	60–79	London	5–10	After school club	8–11 years
	LGC003	Woman	20–39	North West	<1	Youth club	8–17 years
	LGC004	Woman	40–59	North West	10–15	After school club	5–11 years and adults
	LGC005	Man	40–59	London	5–10	During school	3–11 years
	LGC006	Woman	40–59	East	<1	During school	6–11 years
	LGC008	Woman	40–59	West Midland	5–10	During school	7–11 years
Food waste reduction	LGC007	Woman	40–59	North West	<1	Community centre	Adults

LGC, Let’s Get Cooking.

Five key themes were generated and are detailed below; (1) Perceived benefits of LGC; (2) Perceptions of training; (3) Adapting for participant needs; (4) Resourcing clubs; (5) Networking.

#### Perceived benefits of LGC

Participants expressed belief in the need for LGC due to an observed lack of cooking skills and knowledge in both children and adults. Clubs were described as *‘very popular’*, with high participant demand and support from caregivers. Perceived benefits of LGC included acquisition of cooking and food skills, with some participants described as lacking the *‘basics’*, psychosocial benefits (e.g. social skill development) and exposure to new foods. ‘Food waste reduction’ programme participants were described as learning practical waste reduction strategies and generating less food waste.

*‘It’s providing a lot of enjoyment, a lot of education. Do you know what I mean? It’s it’s put to a really good use. So I can’t see why it shouldn’t long continue’ (LGC007)*

Replication of skills outside sessions was mentioned for both programmes. The extension of skills, such as batch cooking, to families in ‘school and community-based’ clubs was described, although in most cases parental attendance was at special events rather than regular sessions.

#### Perceptions of training

Training is perceived as *‘very informative’*, equipping club leaders with knowledge, skills, *‘confidence’* and useful *‘hints and tips’*. Most advocated for in-person training, believing *‘the more practical, the better’*. Online training was felt to communicate the *‘aims’* and *‘values’* of LGC; however, perceptions of its sufficiency to provide *‘the skills to teach’* children were mixed, and the inclusion of online demonstrations was advocated for,

*‘Where the person like yourself is there delivering the training, perhaps cooking some of the recipes or going through some of the steps’. (LGC008)*

The desire for ongoing training was apparent. Conducting onsite training in schools was suggested to increase the number of staff members available to support programme delivery. Separate *‘refresher’* training for established club leaders was also suggested, focusing on recipe preparation to avoid *‘repetitive’* training.

*‘If I’ve done the training before, I don’t need a lengthy spiel on how to cut…but what I do need is being taught how to cook hot food’. (LGC005)*

#### Adapting for participant needs

LGC delivery ranges in setting and extent of integration into a wider programme (e.g. during a youth club) and was described as evolving over time. Variations in session delivery were described in response to different participant characteristics, including age, behaviour or cultural context. Some felt younger participants require more supervision, demonstration and a *‘simpler, slower…more clearly outlined’* recipe. Others described multi-age sessions as mitigating this challenge as *‘older children can help the younger children’* and facilitating siblings’ attendance by avoiding multiple pick-up times for parents. Adaptations for vulnerable children or those with *‘additional needs’* included delivering in-school sessions to promote inclusion or shortening sessions to maintain engagement.

Generally, LGC resources were perceived as adaptable and suitable for a variety of participants, with the *‘same [resource] being used in a slightly different way’*. However, interviewees described a degree of trial and error to find the *‘recipes that really work for you’* and in some cases supplement resources with additional material. Several clubs including *‘children new to the country’,* non-native English speakers or adults with refugee status described a need for more culturally inclusive recipes, such as Halal or vegetarian. Others described participants’ reluctance to try certain foods as a barrier to preparing LGC’s healthier recipes, creating a need to select or alter recipes to be *‘user friendly’,*

*‘I’d probably changed certain ingredients in the, in the foods, in the recipes…because our kids, won’t eat certain things, so a lot of our kids won’t eat veg’. (LGC003)*

#### Resourcing clubs

Overall club leaders felt *‘well resourced’*, expressing appreciation for LGC’s funding, which facilitates the purchase of equipment and ingredients. However, funding pressures remain and, although not viewed as critical to clubs’ continuation in the near term, some described these increasing due to sporadic financial support, rising ingredient costs and budgetary competition, particularly in schools. Financial self-sufficiency was seen as unlikely and even unattainable in more deprived areas. Discomfort with the possibility of charging participants to cover costs was expressed.

Additional challenges constraining session delivery included limited cooking facilities, facilitator availability and tight session timings. Club leaders described using only the cheapest, quickest recipes, recipes requiring no cooking, reducing session frequency due to being unable *‘to afford to do it every week’* and limiting group size or compromising participant autonomy due to equipment sharing.

*‘Some [recipes] required…resources that we didn’t have, so we couldn’t cook them.’ (LGC005)*

Consequently, the need for budget recipes with estimated costs and fewer ingredients was emphasised, both for clubs and families replicating recipes at home. Furthermore, provision of the equipment required to replicate training recipes was suggested, including a *‘basic cookery pack’* to adequately resource new clubs. Generally, club leaders described resources as *‘really useful’*, making sessions *‘more efficient’*. However, established club leaders felt resource provision has declined, highlighting the ceasing of termly packs, skill progression focused resources and a dedicated LGC website.

#### Networking

Club leaders described the *‘ability to network with other organisations’* as important for providing a sense of *‘belonging’*, a resource for *‘solving problems’* and a platform for knowledge sharing. However, network links were felt to have *‘diminished’* and the desire for greater community connection was expressed.

*‘Those old links are sort of diminished slightly…it would be good to have a session online at some stage, maybe just to recap for everybody, what everybody’s doing and kind of how important it all is’ (LGC001)*

## Discussion

This study investigated the content, delivery and existing evidence base for LGC’s effectiveness to inform future programme design and evaluation refinements, drawing on the ‘planning’ principles outlined in the first three stages of the Cook-Ed^TM^ model^(23)^. The results of past evaluations suggest that LGC’s ‘school and community-based’ programme may improve cooking skills, modestly increase short-term consumption of healthy items^(25–28)^ and deliver psychosocial benefits^(25–28)^, in line with recent evidence suggesting that cooking behaviour may mediate both diet quality and mental health^(33)^, conferring wellbeing^(34)^. These benefits were also described by club leaders, who reported observing cooking and social skill acquisition. Retrospective application of the CALO-RE taxonomy^(21)^ to LGC resources identified the most commonly occurring BCT targeting cooking behaviour across programmes as how (BCT#21) and when (BCT#20) to perform the behaviour, highlighting individual consequences of the behaviour (BCT#2) and prompting practice (BCT#26). The frequent identification of BCT#26 in resources may explain observed positive dietary outcomes in past evaluations, with evidence suggesting a ‘hands-on’ approach can create an ‘IKEA’ effect, whereby self-preparation increases preference for homemade and healthy items^(35)^. Club leaders also expressed their preference for ‘hands-on’, in-person training due to perceived benefits for skill acquisition, as postulated by Experiential Learning Theory^(36)^. Prospective or retrospective application of BCT taxonomies to interventions targeting children remains relatively uncommon^(37)^. Therefore, this study fills a gap both in previous LGC evaluations and the wider literature.

In aggregate, LGC’s ‘school and community-based’ recipes contain cooking skills appropriate for all participating children (aged from 3 to 17 years); however, recipes did not specify appropriate age ranges. Subjective assessment of additional aspects related to age-appropriateness highlighted that the layout and language of recipes may not be child-friendly; small fonts and lack of visual aids may further limit resource accessibility. Club leaders reported adapting resources in response to participant needs, including making age-related simplifications. LGC’s ‘train the trainer’ model was seen as providing this necessary flexibility and previous LGC evaluations suggest this delivery model may be effective, potentially due to increased personal relevance to participants, increasing the likelihood of behaviour change^(22)^. However, due to a lack of assessment of programme ‘fidelity’ and ‘dose delivered’ in previous evaluations, the extent to which these variations in delivery may impact observed outcomes is currently unknown. ‘Food waste reduction’ programme resources primarily include strategies relating to ‘food literacy, kitchen tools and equipment’ and ‘cooking and leftover reuse’^(30)^. Anecdotal interview evidence in this study provides positive indications that learning practical waste reduction strategies through participation in LGC may result in a reduction in food waste generation; however, this has not been formally evaluated.

The findings of this study highlight several areas for consideration at the ‘development and implementation’ phase of refinement^(23)^. Deconstruction of resources demonstrated overlap between the most frequently occurring BCTs in the ‘school and community-based’ and ‘food waste reduction’ programmes, despite different target age groups. Culinary interventions targeting adult populations also share frequently occurring BCT (BCT#21 and 26)^(19)^, suggesting that they may target cooking behaviour independently of age. This may be a strength of LGC’s programme design as club leaders describe delivery in both single and multi-age groups, including to adults and children together. Given delivery of LGC can be to children across multiple age groups, it could be argued that distributing recipes targeted at single age groups is not appropriate or that the variable impact of individual development trajectories and willingness to try^(29)^ may negate the need for skill focused adaptations. However, as numeracy and literacy skills are required for skill acquisition and reading recipes^(29)^, the lack of step-by-step instructions on how to perform certain cooking skills and the complex language used in the methods section of LGC recipes may hinder children’s skill acquisition. This could be both directly, through lack of understanding, or indirectly, from associated negative feelings of embarrassment. Therefore, redesign of LGC’s child-facing resources to accommodate a range of literacy levels may enhance children’s cooking skill acquisition^(29)^. This could include simplifying recipe layouts, breaking down cooking skills, enlarging font and providing visual aids to increase accessibility. Provision of separate club leader-facing recipes which indicate the age-appropriateness of included skills may enable them to make more informed recipe selections.

This study identified several opportunities to support club leaders delivering LGC. Firstly, interviews suggest a proportion of LGC’s recipes are not fit for purpose considering the resource and budgetary constraints facing both clubs and participants. Recipes were also felt to lack cultural diversity. If recipes covered in sessions are not replicable in participants’ home environment, this could contribute to an ‘intention-behaviour gap’^(38)^, limiting meaningful long-term behaviour change. Additionally, time constraints highlighted by club leaders may limit their capacity to fully adapt programme resources to meet participants’ diverse needs, potentially reducing LGC’s efficacy. Therefore, it is important to consider external factors, including cost and access to cooking facilities, in programme design. Greater provision of club leader-facing resources, including recipe cost estimates and expanding the cultural inclusivity of provided recipes, may support delivery. Consulting key stakeholders (club leaders, participants) regarding future resource adaptations will be important to ensure acceptability for end users. Secondly, club leaders expressed a desire for ongoing training. Online training is likely less time and resource-intensive, however, and may limit opportunities for social modelling^(39)^, inhibiting acquisition of the necessary skills for session delivery, a concern raised by some club leaders. An alternative approach may be the introduction of supplementary instructional video material, with evidence suggesting step-by-step video instructions may improve understanding of cooking methods by reducing cognitive load^(40)^. Further investigation into the potential for an integrated online and in-person training approach is required and may have broader public health applications. Finally, interviews demonstrate club leaders’ desire for knowledge sharing and a greater sense of community. Beyond the direct implication of strengthening LGC’s inter-club network, embedding co-design in programme refinements may offer mutual benefit in building community links while leveraging club leaders’ experience to increase programme acceptability^(17,23)^. Investigating the perspectives of programme stakeholders through interviews is a strength of this study forming part of the ‘planning’ phase of programme refinement^(23)^. However, co-design can require time and financial investment and so alternative formats, such as focus groups, could be explored in future.

Deconstruction of ‘food waste reduction’ resources suggests the programme may target several predictive factors of intention to reduce food waste^(41)^. Resources include information on how (BCT#21) and when (BCT#20) to perform behaviours which may increase competence and perceived behavioural control^(41)^, reinforced by advice on environmental restructuring (BCT#24) or action planning (BCT#7). Descriptions of the individual (BCT#2) and general (BCT#1) consequences of food waste in these resources may target attitudes and anticipated regret, invoking concern for doing the ‘right thing’^(42)^. ‘Food waste reduction’ programme resources contain dual messaging concerning food waste reduction and healthier eating, primarily promoting food preparation from scratch. However, this may introduce conflicting behavioural drivers as food waste concerns can act as a barrier to cooking from scratch^(43)^, while a desire to offer healthy foods may lead to overprovision, increasing waste^(6)^. A full programme evaluation is required to elucidate the programme’s relative impact on target healthy and sustainable behaviours. Parallel acquisition of cooking and food waste reduction skills may reduce the perceived conflict between these behaviours, offering an alternative mechanistic interpretation of the association between early cooking skill acquisition and food waste reduction^(8)^. Recent frameworks for culinary intervention design incorporate sustainable food practices^(17)^; however, this is not currently reflected in the ‘school and community-based’ programme. Integrating age-appropriate food waste education into sessions for children could help foster sustainable practices and a pro-environmental self-identity^(42)^.

Assessment of past evaluations has identified evidence gaps and methodological limitations for future consideration. Process evaluation is critical to understand and monitor the implementation of a programme ahead of assessing its effectiveness^(23)^. However, it could be argued that within a ‘train the trainer’ model, a single definition of acceptable programme delivery, and by extension inclusion of ‘fidelity’ and ‘dose delivered’, unaddressed by previous evaluations, is redundant. Conversely, this may increase the importance of understanding which components of session delivery are critical to LGC’s effectiveness, which are adaptable without compromising effectiveness, and if/how contextual factors and challenges (e.g. lack of facilities, funding or time) impact this. Additionally, mapping how BCT included in refined programme design are reflected in implementation will help understand which components are associated with positive outcomes. Cooking skills and diet quality have a non-uniform relationship with sociodemographic characteristics^(3,5,11)^. However, previous evaluations of LGC’s ‘school and community-based’ programme did not present analysis of socioeconomic factors; thus, it cannot be assumed that reported outcomes are consistent across sub-groups. Given the diversity of LGC participants, analysis of sociodemographic variables would strengthen future evaluations. Impact evaluation in both adults and children should prioritise the use of validated instruments to assess cooking behaviour influences, such as CooC7 and CooC11 for children’s cooking confidence^(44)^ or the ‘checklist’ for food skill acquisition in adults^(45)^. Ensuring future impact evaluations are adequately powered would increase the reliability of findings. Finally, culinary interventions such as LGC can be ‘resource-intensive’^(23)^, with a paucity of data reporting programme costs^(13)^. Therefore, assessments of cost-effectiveness may be required to understand the programme’s ongoing economic viability.

### Strengths and limitations

This study is the first evaluation of LGC conducted since 2017. Strengths of this research include examination of the totality of publicly available evaluations of LGC since its inception in 2007. However, synthesis was conducted ‘outside in’, without access to raw data or full evaluation methodologies, reducing the reliability of results. Resources deconstructed reflect all those available for a given programme and type or were confirmed as representative with the BDA, increasing the generalisability of results across LGC. However, due to time constraints, in some cases a random selection of resources was deconstructed; deconstruction of the entirety of LGC’s programme resources would have strengthened this study. Deconstruction of resources using published theoretical frameworks increases comparability and repeatability of results. However, frameworks were not designed for retrospective application and analysis is subject to inherent limitations within the frameworks themselves. Due to time constraints, the primary coder received no formal BCT coding training, which is a skilled task^(46)^; however, a high level of inter-rater reliability with a second trained coder was reached. Utilising quantitative and qualitative approaches within a mixed methods design increases confidence in the findings from respective approaches. Given narrower study aims and a high dialogue quality, the interview sample size (*n* 7) was deemed to generate sufficient information power^(47)^. Interview questions were open-ended; however, participants’ answers may be susceptible to social-desirability bias. Convenience sampling increases the risk of self-selection bias and only one food waste programme interview was conducted. However, a range of regions and club maturities were sampled, with no reason to believe results are not representative of club leaders generally. Use of focus group interviews in future research to promote discussions between club leaders may strengthen findings to inform programme refinements.

## Conclusions

Previous evaluations suggest LGC may be an effective intervention for disseminating cooking and food skills, modestly improving healthy food consumption. However, understanding of which programme components contribute to effectiveness is limited by a lack of reported ‘fidelity’ and ‘dose delivered’ assessment. Identification of BCT suggests that LGC resources target behaviour change through describing how and when to perform cooking behaviour, highlighting individual consequences of the behaviour and prompting practice. ‘School and community-based’ resources contain age-appropriate cooking skills; however, adapting child-facing resources may improve accessibility for children (e.g. larger font, visual aids). LGC’s ‘food waste reduction’ programme primarily includes strategies relating to food management skills and results highlight potential for incorporating food waste reduction elements into the ‘school and community-based’ programme. LGC training is well received, and ongoing training and networking opportunities are desired. The primary benefit of LGC’s ‘train the trainer’ model is the ability to meet different clubs’ needs, although limited funding, facilities and time are challenges in session delivery. Resource adaptations, such as age specifications and cost estimations, may support club leaders in facing these challenges. Further rigorous evaluation is required to better understand LGC’s effectiveness, as well as the impact of contextual challenges and variations in programme delivery on observed outcomes. Future evaluation should include analysis of relevant socioeconomic variables to elucidate sub-group differences, ensure validated measurement instruments are utilised when assessing programme effectiveness and consider incorporating co-design elements such as qualitative interviews and focus groups. Improved understanding of programme adaptations and effectiveness within different sub-groups may have wider public health implications.

## Supporting information

10.1017/S1368980026102675.sm001 Browne et al. supplementary material 1 Browne et al. supplementary material

10.1017/S1368980026102675.sm002 Browne et al. supplementary material 2 Browne et al. supplementary material

10.1017/S1368980026102675.sm003 Browne et al. supplementary material 3 Browne et al. supplementary material

10.1017/S1368980026102675.sm004 Browne et al. supplementary material 4 Browne et al. supplementary material

10.1017/S1368980026102675.sm005 Browne et al. supplementary material 5 Browne et al. supplementary material

10.1017/S1368980026102675.sm006 Browne et al. supplementary material 6 Browne et al. supplementary material

## Supplementary material

For supplementary material accompanying this paper, visit https://doi.org/10.1017/S1368980026102675

## Table 1. Long description

The initial five-year evaluation used self-report participant questionnaires (children key stage 2 and over) or self-complete picture card exercise (children key stage 1 and under), qualitative feedback (club leaders, participants, caregivers); and online activity reports. Total reach: 1.8 million people (average age 9.7 years). Over 90% of participants replicated skills at home (81% involving family/friends), 58% reported improvement in ‘healthy’ item consumption. Evaluations in ‘looked after children’ used participant cooking journal and focus groups with carers. 137 carers/staff were trained. Majority of provided recipes were replicated, 86% of children tried new foods. Evaluation in ‘schools-based clubs’ used online feedback forms, surveys and interview with club leaders. Total read: c.159k children, c.24k adults. Reported improvement in cooking skills, hygiene awareness and trying new foods. Psychosocial benefits reported in all evaluations.

Navigate back to Table 1..

## Table 2. Long description

The table shows the resource type by programme and the deconstruction processes undertaken for each resource. A total of 10 resources were deconstructed from the ‘school and community-based programme’ including for ‘session delivery’ (N=4), ‘session delivery including recipes’ (N=3) and ‘educational’ (N=3) resources. All ‘school and community-based’ programme resources underwent BCT mapping and ‘session delivery including recipes’ resources underwent age-appropriateness assessment. A total of 10 resources were deconstructed from the ‘food waste reduction’ programme including ‘session delivery’ (N=3), ‘educational’ (N=4) and ‘educational including recipes’ (N=3) resources. All ‘food waste reduction’ programme resources underwent BCT mapping and 6 resources underwent food waste reduction strategy indentification (1 ‘session delivery’, 2 ‘educational’ and 3 ‘educational including recipes’).

Navigate back to Table 2..

## Table 3. Long description

The table shows the 18 BCTs which were identified across LGC programme resources. In order of most to least frequently occuring these, with total % occurrence in resources, were: BCT#21 Provide instructions on how to perform the behaviour (95%), BCT#20 provide information on where and when to perform the behaviour (65%), BCT#2 give information on individual consequences (65%), BCT#26 prompt practice (55%), BCT#1 give information on general consequences (50%), BCT#22 model/demonstrate behaviour (40%), BCT#15 prompt generalisation of behaviour (35%), BCT#4 provide normative information (30%), BCT#5 goal setting (10%), BCT#8 barrier identification/problem solving (25%), BCT#18 prompt focus on past success (25%), BCT#10 prompt goal review (10%), BCT#12 prompt reward of effort/progress (10%), BCT#13 prompt reward of success (10%), BCT#29 plan social support/change (10%), BCT#30 prompt identification as role model (10%), BCT#24 environmentla restructuring (5%), BCT#7 action planning (5%)

Navigate back to Table 3..

## Figure 1. Long description

Cooking skills are grouped by appropriate age group: 2-3 years (washing fruit/vegetables, kneading/mixing with hands, tearing, using a rolling pin, rolling mixtures into balls, breaking vegetables into pieces, using a cooking cutter), 3-5 years (stirring/mixing, mashing, spooning, cutting chopping and slicing, spreading/buttering, using scissors, brushing oil on with a pastry brush, sieving, squeezing), 5-7 years (sprinkling and rubing in, breading flouring and dipping, picking and podding, greasing, peelign with fingers, skewering, pouring from a container, crusing and pounding, shaking liquids in a sealed container), 7-9 years (weighing and measuring, using a grater, using an oven or microwave, draining, using a hand mixer) and 9+ years (using a peeler, using a can opener, stirring and mixing over heat, using sharp scissors, unsupervised skewering, weighing, measuring grating, using an oven or microwave unsupervised, using a hand mixer unsupervised)

Navigate back to Figure 1..

## Figure 2. Long description

The total and percentage occurrence of food waste reduction strategies in 6 deconstructed resources were: store food appropriately (N=8,67%), prepare food appropriately (N=8,67%), assess food edibility (N=7,83%), systematically store and label foods (N=6,50%), freeze leftovers (N=5,83%), repurpose surplus food (N=5,50%), serve and store accurate portions (N=5,83%), repurpose leftovers (N=4,67%), use store cupboard ingredients (N=3,50%), freeze ingredients (N=3,50%), collect storage equipment (N=3,50%), prepare meals in advance (N=2,33%), batch cook (N=2,33%), compost surplus food (N=1,17%), meal plan (N=1,17%), create a shopping list (N=1,17%), keep a food inventory (N=1,17%), stock take-away alternatives (N=1,17%), purchase marked down products (N=1,17%), economise on staples (N=1,17%), serve common meals (N=1,17%), prioritise near-expired products (N=1,17%).

Navigate back to Figure 2..

## Table 4. Long description

6 participants were club leaders of ‘school and community-based’ clubs (LGC001,3,4,5,6,8) and 1 was a club leader of a ‘food waste reduction’ club (LGC007). Details by participant: LGC001: woman aged 60-79 years; club details: after school for 8-11 year olds; London; maturity: 5-10 years. LGC003: woman aged 20-39 years; club details: youth club for 8-17 year olds; North West England; maturity: >1 year LGC004: woman aged 40-59 years; club details: after school for 5-11 year olds & adults; North West England; maturity 10-15 years. LGC005: man aged 40-59 years; club details: during school for 3-11 year olds; London; maturity: 5-10 years. LGC006: woman aged 40-59 years; club details: during school for 6-11 year olds; East England; maturity: >1 year LGC007: woman aged 40-59 years; club details: community centre for adults; North West England; maturity: >1 year. LGC008: woman aged 40-59 years; club details: during school for 7-11 year olds; West Midlands; maturity 5-10 years.

Navigate back to Table 4..
