# Supplementary material for: Let’s Get Cooking: a mixed methods evaluation of a programme delivering culinary education via a network of cookery clubs across England
Source: Public Health Nutr. 2026 May 21;29(1):e126. doi: 10.1017/S1368980026102675 (PMC13434492; doi:10.1017/S1368980026102675)
Supplement: Supplementary file 1 [file S1368980026102675sup001.docx]

**Supplementary Material A**

***Additional Programme Details – Evaluation Methodologies, Programme Aim and Design***

| **Table A.1.**  Sampling and evaluation methodologies used in previous evaluations of ‘Let’s Get Cooking’ (LGC) | | | |
| --- | --- | --- | --- |
|  | **Five-year ‘Evaluation of the Let’s Get Cooking Programme: Final Report’**^(1)^**and Summary**^(2)^**2012** | **Let’s Get Cooking: School-based Clubs (2015-2017) Report Summary**^(3)^ | **‘Cooking with Looked After Children: Evaluation Report’ 2016**^(4)^ |
| **Sampling Methodology** | - Representative, random, stratified sample of 271 clubs (5.5% of total), of which 243 completed full evaluations following exclusions (e.g. clubs with only infant participants) - Sampling carried out on a rolling basis from year one (2008) to year five (2012) | - Convenience sampling: all club leaders asked to participate in reporting - Sampling of schools for interview not reported | - Convenience sampling: all carers and children asked to participate |
| **Sample** | **Evaluation Methodology** | | |
| **Programme Leaders & Facilitators** | - Post-training feedback forms - Routine online activity reports completed by club leaders throughout LGC programmes - Additional qualitative feedback collected by field researchers was coded to determine the most popular responses | - Post-training feedback forms (N=2,071) - Routine online termly feedback forms - Annual club survey in 2016 and 2017 (N=1,123) - 14 interviews conducted in 11 primary schools | - Post-training feedback forms from carers and residential children’s home staff (N=126) - Focus groups (N=15) |
| **Participants – Children (above KS1)** | - Evaluation conducted at baseline, programme conclusion (N=1,740-2,433) and at 3-month follow up  (N=357) - Self-complete questionnaire and tick list of 52 items, including ‘healthy’, ‘unhealthy’ and ‘neutral’ items, from which a ‘healthy food score’ was generated by subtracting the number of unhealthy items from the number of healthy items. A more positive score indicated healthier dietary intake - Additional qualitative feedback collected by field researchers was coded to determine the most popular responses | n/a | - Cooking journals completed throughout programme  (N = 108) |
| **Participants – Infants (KS1 and below)** | - Self-complete individual picture card exercise at baseline and programme conclusion, identifying healthy foods eaten and drunk (N=335). No timeframe was specified. - Additional qualitative feedback collected by field researchers was coded to determine the most popular responses |  |  |
| **Parents and Caregivers** | - Qualitative feedback collected by field researchers |  | (see programme facilitators) |

| **Table A.2.**:  Further programme details provided in previously published LGC evaluations | | | |
| --- | --- | --- | --- |
|  | **Five-year ‘Evaluation of the Let’s Get Cooking Programme: Final Report’** ^(1)^**and Summary** ^(2)^**2012** | **Let’s Get Cooking: School-based Clubs (2015-2017) Report Summary** ^(3)^ | **‘Cooking with Looked After Children: Evaluation Report’ 2016** ^(4)^ |
| **Funding** | - Big Lottery Fund (£20 million) | - Tesco Eat Happy Project (amount not specified) | - Iceland Charitable Foods Foundation (amount not specified) |
| **Target Population** | - Children and young people aged 5 to 18, their family members and members of the local community - Schools in ‘disadvantaged areas’, with high levels of free school meal eligibility prioritised | - Primary school aged children - Clubs prioritise those who are, or have been, either eligible for free school meals and/or looked after by local authority or state care, and children with special educational needs | - Looked after children in foster care or residential children’s homes |
| **Programme Aims and Objectives** | - To increase the food preparation or cooking skills of 1,106,300 children, family members and members of the local community - For 70% of club members who learn a new healthy eating skill to replicate that skill at home - For 50% of club members to increase their intake of nutritionally healthy food | - Specific, measurable ingoing aims not defined | - Specific, measurable ingoing aims not defined - Report states the programme’s purpose ‘to equip [carers] with the skills and confidence to cook with the children in their care and help those children cook and eat more healthily’ |
| **Training Delivered** | - Facilitators of new clubs received practical training, including an introductory session, ‘start-up day’ and two-day demonstrator course accredited by the Royal Society of Public Health - Facilitators of both new and associate clubs accessed top up training and regular LGC conferences - Topics covered in training included ‘cookery demonstration skills’, ‘food safety, nutrition and health and safety’ | - Training of 2,662 club leaders delivered in nine regions across England - Year one training delivered as a ‘conference-style theory-based event’ with a choice of workshops - Year two training revised in line with feedback to be delivered as ‘smaller, more practical based events’ including practical cooking and educational sessions - Topics covered included the Eatwell guide, the ‘implications of the Childhood Obesity Plan’, food and allergen labelling, nutrition, health and safety, and how to raise funds and awareness for clubs | - Delivered to 137 foster carers and residential staff across 8 cities in the UK - Included practical cooking, group discussion and sessions on ‘healthy eating’, ‘cooking on a budget’, ‘creating and making use of store cupboard ingredients’ and ‘freezing, batching and storing food’ |
| **Programme**  **Design** | - Sessions delivered by teachers, school cooks, parents or other community volunteers - During sessions, participants prepared a healthy recipe from ‘basic ingredients’ and were given a copy to encourage replication at home - A skills progression approach was used with charts to measure progress in cooking skills such as ‘weigh and measure’ - Participants were encouraged to be ‘ambassadors’ in their community, sharing their skills - Resources provided included recipes, activity packs and ongoing support from LGC staff - Clubs were provided funding for ingredients and equipment but supported towards becoming self-sustaining | - Delivery of four cooking sessions per term - Resources provided included a recipe book, session plan and other resources such as newsletters - £50 Tesco gift card provided towards cost of ingredients and equipment | - Carers delivered 10 sessions to children in their care in small groups - Resources provided included a recipe book and funding towards ingredient and equipment costs |

**References**

1.	School Food Trust (2012) *Evaluation of the Let’s Get Cooking programme. Final Report*. .

2.	Children’s Food Trust & School Food Trust (2011) *A recipe for healthier communities: The impact of Let’s Get Cooking*. .

3.	Children’s Food Trust (2017) *Let’s Get Cooking: School-based Clubs (2015-2017) Report summary*. .

4.	Children’s Food Trust (2016) *Cooking with looked after children. Evaluation report for Iceland Foods Charitable Foundation*. .
