# Supplementary material for: Let’s Get Cooking: a mixed methods evaluation of a programme delivering culinary education via a network of cookery clubs across England
Source: Public Health Nutr. 2026 May 21;29(1):e126. doi: 10.1017/S1368980026102675 (PMC13434492; doi:10.1017/S1368980026102675)
Supplement: Supplementary file 2 [file S1368980026102675sup002.docx]

**Supplementary Material B**

***Inter-rater Reliability***

The breakdown of inter-rater reliability by resource is shown in Table B.1. Percentage agreement for inclusion and frequency of occurrence by behaviour change technique (BCT) was calculated on a ‘yes/no’ basis, as demonstrated by the exemplar in Table B.2.

| **Table B.1.**  Intercoder reliability by resource | | | | |
| --- | --- | --- | --- | --- |
| **Deconstruction Process** | **Resource** | **Programme** | **Percentage Agreement** | |
|  |  |  | **BCT Inclusion** | **Frequency of Occurrence** |
| Food Waste Prevention Strategy Identification | Freezing, Batching, Storing | Food waste reduction | 87% | 73% |
| BCT Mapping | Guide to Running Cooking Sessions | School and community-based | 93% | 64% |
|  | Make the Most of Your Mince | Food waste reduction | 100% | 80% |
| Age-appropriateness Assessment | Oriental Turkey Mince Boats* | School and community-based | 100% | 77% |
|  | Mini Veggie Pizzas* |  | 100% | 88% |
| Average | | | 95% | 77% |
| *Recipe from ‘Favourite Takeaways to Cook at Home’ resource | | | | |

| **Table B.2.**  Example intercoder reliability calculations - ‘Make the Most of Your Mince’ | | | | | |
| --- | --- | --- | --- | --- | --- |
| **BCT** | | **BCT Occurrence by Coder** | | **Intercoder Agreement?** | |
|  |  | **FL** | **GB** | **BCT Inclusion** | **Frequency of Occurrence** |
| 20 | Provide information on where and when to perform the behaviour | 6 | 6 | yes | yes |
| 2 | Provide information on consequences of behaviour to the individual | 1 | 1 | yes | yes |
| 1 | Provide information on consequences of the behaviour in general | 2 | 1 | yes | no |
| 26 | Prompt practice | 1 | 1 | yes | yes |
| 21 | Provide instruction on how to perform the behaviour | 8 | 8 | yes | yes |
| Percentage agreement | | | | 100% | 80% |
