# Supplementary material for: Let’s Get Cooking: a mixed methods evaluation of a programme delivering culinary education via a network of cookery clubs across England
Source: Public Health Nutr. 2026 May 21;29(1):e126. doi: 10.1017/S1368980026102675 (PMC13434492; doi:10.1017/S1368980026102675)
Supplement: Supplementary file 3 [file S1368980026102675sup003.docx]

**Supplementary Material C**

***Definitions of Behaviour Change Techniques***

| **Table C.1.**  List of BCTs and definitions included in the ‘Coventry, Aberdeen & London – Refined’ (CALO-RE) taxonomy | |
| --- | --- |
| **BCT Number** | **Definition** |
| BCT#1 | Provide information on consequences of the behaviour in general |
| BCT#2 | Provide information on consequences of behaviour to the individual |
| BCT#3 | Provide information about others’ approval |
| BCT#4 | Provide normative information about others’ behaviour |
| BCT#5 | Goal setting (behaviour) |
| BCT#6 | Goal setting (outcome) |
| BCT#7 | Action planning |
| BCT#8 | Barrier identification / problem solving |
| BCT#9 | Set graded tasks |
| BCT#10 | Prompt review of behavioural goals |
| BCT#11 | Prompt review of outcome goals |
| BCT#12 | Prompt rewards contingent on effort or progress towards behaviour |
| BCT#13 | Provide rewards contingent on successful behaviour |
| BCT#14 | Shaping |
| BCT#15 | Prompting generalisation of a target behaviour |
| BCT#16 | Prompt self-monitoring of behaviour |
| BCT#17 | Prompt self-monitoring of behavioural outcome |
| BCT#18 | Prompting focus on past success |
| BCT#19 | Provide feedback on performance |
| BCT#20 | Provide information on where and when to perform the behaviour |
| BCT#21 | Provide instruction on how to perform the behaviour |
| BCT#22 | Model/Demonstrate the behaviour |
| BCT#23 | Teach to use prompts/cues |
| BCT#24 | Environmental restructuring |
| BCT#25 | Agree behavioural contract |
| BCT#26 | Prompt practice |
| BCT#27 | Use of follow-up prompts |
| BCT#28 | Facilitate social comparison |
| BCT#29 | Plan social support/social change |
| BCT#30 | Prompt identification as role model/position advocate |
| BCT#31 | Prompt anticipated regret |
| BCT#32 | Fear arousal |
| BCT#33 | Prompt self-talk |
| BCT#34 | Prompt use of imagery |
| BCT#35 | Relapse prevention/coping planning |
| BCT#36 | Stress management/emotional control training |
| BCT#37 | Motivational interviewing |
| BCT#38 | Time management |
| BCT#39 | General communication skills training |
| BCT#40 | Stimulate anticipation of future rewards |
