# Supplementary material for: Let’s Get Cooking: a mixed methods evaluation of a programme delivering culinary education via a network of cookery clubs across England
Source: Public Health Nutr. 2026 May 21;29(1):e126. doi: 10.1017/S1368980026102675 (PMC13434492; doi:10.1017/S1368980026102675)
Supplement: Supplementary file 4 [file S1368980026102675sup004.docx]

**Supplementary Material D**

**Semi-structured interview procedure**

Background information

- Do you mind answering a few background questions so that we can understand a bit about you and your experience within the LGC programme?
- Have you had any previous experience of health / nutrition promotion work?
- What sort of groups were you running LGC clubs for?
- How many people would you have in each session?
- What kind of participants?

Views about training delivered

- What are your overall impressions of the training delivered by LGC? (5 mins)
- How did the training impact the way you run the club? (5 mins)
- Are there any further topics you would like further training on? (1-2 mins)
- Do you have any suggestions on how the training could be improved? (1-2 mins)

Views about running a programme

- What did you think about when planning a club series and sessions?
- Has the way that you run your club changed at all over time?
- Have you faced any challenges running an LGC club?
- What is your perspective on the aim for LGC clubs to become self-sufficient?
