# Supplementary material for: Let’s Get Cooking: a mixed methods evaluation of a programme delivering culinary education via a network of cookery clubs across England
Source: Public Health Nutr. 2026 May 21;29(1):e126. doi: 10.1017/S1368980026102675 (PMC13434492; doi:10.1017/S1368980026102675)
Supplement: Supplementary file 5 [file S1368980026102675sup005.docx]

**Supplementary Material E**

***Consolidated criteria for reporting qualitative research (COREQ): 32-item checklist***

| **Table E.1.**  Completed COREQ checklist for qualitative research conducted in the study of ‘club leaders’ perspectives on LGC’ | | | |
| --- | --- | --- | --- |
| **Item No** | | **Guide Questions/Description** | **Reference Manuscript Section** |
| ***Domain 1: Research team and reflexivity*** | | | |
| *Personal Characteristics* | | | |
| 1. Interviewer/ facilitator | | Which author/s conducted the interview or focus group? | 2.3 |
| 2. Credentials | | What were the researcher’s credentials? E.g., PhD, MD | 2.3 |
| 3. Occupation | | What was their occupation at the time of the study? | N/A |
| 4. Gender | | Was the researcher male or female? | 2.3 |
| 5. Experience and training | | What experience or training did the researcher have? | 2.3 |
| *Relationship with participants* | | | |
| 6. Relationship established | | Was a relationship established prior to study commencement? | 2.3 |
| 7. Participant knowledge of the interviewer | | What did the participants know about the researcher? e.g. personal goals, reasons for doing the research? | 2.3 |
| 8. Interviewer characteristics | | What characteristics were reported about the interviewer/facilitator? e.g. Bias, assumptions, reasons and interests in the research topic | 1.0; 2.3 |
|  | ***Domain 2: study design*** | | |
|  | *Theoretical framework* | | |
|  | 9. Methodological orientation and Theory | What methodological orientation was stated to underpin the study? e.g. grounded theory, discourse analysis, ethnography, phenomenology, content analysis | 2.3 |
|  | *Participant selection* | | |
|  | 10. Sampling | How were participants selected? e.g., purposive, convenience, consecutive, snowball | 2.3 |
|  | 11. Method of approach | How were participants approached? e.g., face-to-face, telephone, mail, email | 2.3 |
|  | 12. Sample size | How many participants were in the study? | 3.3 |
|  | 13. Non-participation Setting | How many people refused to participate or dropped out? Reasons? | 3.3 |
|  | 14. Setting of data collection | Where was the data collected? e.g., home, clinic, workplace | 2.3 |
|  | 15. Presence of nonparticipants | Was anyone else present besides the participants and researchers? | 2.3 |
|  | 16. Description of sample | What are the important characteristics of the sample? e.g. demographic data, date | 3.3 - Table 4 |
|  | *Data collection* | | |
|  | 17. Interview guide | Were questions, prompts, and guides provided by the authors? Was it pilot tested? | 2.3 |
|  | 18. Repeat interviews | Were repeat interviews carried out? If yes, how many? | 2.3 |
|  | 19. Audio/visual recording | Did the research use audio or visual recording to collect the data? | 2.3 |
|  | 20. Field notes | Were field notes made during and/or after the interview or focus group? | 2.3 |
|  | 21. Duration | What was the duration of the interviews or focus group? | 2.3 |
|  | 22. Data saturation | Was data saturation discussed? | 4.2 |
|  | 23. Transcripts returned | Were transcripts returned to participants for comment and/or correction? | 2.3 |
|  | ***Domain 3: analysis and findings*** | | |
|  | *Data analysis* | | |
|  | 24. Number of data coders | How many data coders coded the data? | 2.3 |
|  | 25. Description of the coding tree | Did the authors provide a description of the coding tree? | N/A |
|  | 26. Derivation of themes | Were themes identified in advance or derived from the data? | 2.3 |
|  | 27. Software | What software, if applicable, was used to manage the data? | 2.3 |
|  | 28. Participant checking | Did participants provide feedback on the findings? | 2.3 |
|  | *Reporting* | | |
|  | 29. Quotations presented | Were participant quotations presented to illustrate the themes/findings? Was each quotation identified? e.g., participant number | 3.3 |
|  | 30. Data and findings consistent | Was there consistency between the data presented and the findings? | 3.3 |
|  | 31. Clarity of major themes | Were major themes clearly presented in the findings? | 3.3 |
|  | 32. Clarity of minor themes | Is there a description of diverse cases or a discussion of minor themes? | 3.3 |
