# Supplementary material for: Let’s Get Cooking: a mixed methods evaluation of a programme delivering culinary education via a network of cookery clubs across England
Source: Public Health Nutr. 2026 May 21;29(1):e126. doi: 10.1017/S1368980026102675 (PMC13434492; doi:10.1017/S1368980026102675)
Supplement: Supplementary file 6 [file S1368980026102675sup006.docx]

**Supplementary Material F**

**Extracts by Theme for Section 3.3. Club Leaders’ Perspectives on LGC**

***3.3.1. Perceived benefits of LGC***

*“Historically, a lot of the children that I'd work with would have experience of cooking at home, so when they came to the cookery club they would have some some knowledge. And so you could progress quite quickly. What I've discovered I would say in the last five years is the children that I'm having now do very little cooking at home. So you having to, like, teach them how to crack an egg, how to whisk an egg. Whereas historically, you'd have children who know how to do that, across the school, little ones right up to big ones, whereas there is less children now who've got that. You, you get the odd one or two or say yes, I cook at home, I make a cake and stuff, but a lot of the children now don't have the basics, you know, they won't know how to grate cheese. They don't know the names of the equipment, so if you say to them, you know, can you go and get this out of the cookery they haven't got a clue what that is. So you have to teach, you know, how to how to juice a lemon, how to, all of that has to be incorporated into the sessions. Yeah, that's something I've really, really noticed. Yeah. Yeah.” (LGC008)*

*“But obviously the parents are delighted by it. You know it all. They they love their children being able to do extracurricular activities. So they do, they are very, very appreciative” (LGC006)*

*“We know the children that would really like it and it's a very popular club. You could kind of fill it a couple of days a week probably” (LGC001)*

*“A lot of our demographic ethnic background for our school is 86% Bangladeshi and I know with Bangladeshi families, parents do everything for their children and. I'm Indian, so I've grown up in, in a sort of similar environment and my mom did everything for me we weren't allowed to do cooking with our parents. It was get out of the kitchen, you know there wasn't that ‘Oh, come and join me and come and help me chop’…So we're really honed in on that, the peeling, the cutting, the chopping, how to use chopping boards, health and hygiene, washing their hands, take, you know, taking the jewellery off, tying your hair back, make sure you've got an apron on all that sort of stuff is is what we would would focus in on from the training” (LGC005)*

*“And then the one to ones when I do it, I have my small coaching group and I'll generally do that on my own with the children so I can hone in on them and see what their, some of the issues are also in the class and we can work together just to use it as a base for talking and for listening and building their self esteem and confidence up in in a different way” (LGC005)*

*“there's some children who find it really find eating difficult for different reasons, so they don't like eating other people or they don't like trying new foods. They've got very, very restricted diets, some of them who are in it. So. So it's just really nice if we sort of sit all sit down together and just eat it together, eat a little bit of it and then they can take some home as well.” (LGC006)*

*“Children the some of the children from our autism and global delayed provision. They will do the cookery club without one to one support because actually it's something that they really like and they really enjoy doing so they can put, they participate and then there's other children that may be known to social services, social care that I will place in just so they have a club and I know that I'm the club leader kind of so I know some of those children.” (LGC001)*

*“There hasn't been this year, but previously we have because we've we've done things like where the children have done their skills progression and then at the end we've had like a celebration event where they've sort of invited their parents in and cooked for them or shared. I mean one year, going back probably about four years ago, we we had a group of children who were really, really good and they actually served their parents a three-course meal at the end, they actually came in and we set it up as a restaurant. And they cooked, waited, did hosting, waitressing skills and all of that, and and did a big celebration at the end” (LGC008)*

*“We're more concerned about waste, about food waste. There's no waste, no more. Do you know what I mean? And as well as like the women now, because everybody's thinking about it, they’re bringing stuff in and and trying things at home now. You know, when you just have got that odd carrot, you might have a tomato. I like, there’s like a lot of brain brain busting together with us, like saying we could do this, we could do that. So yeah, we did learn a lot from it. And it's like especially the waste and like how much you do waste. So what you can do with what you've got” (LGC007)*

*“I liked, as I said before, the idea of the waste food, you know, to have to use it so you're not wasting anything. I thought that was superb…so when there's food extra, we will use that and incorporate into a different meal. So nothing, nothing goes to waste anymore.” (LGC003)*

*“We've got more adventurous. So what we do now, like what I said to you before is, now where a lady from another country, she will actually discuss with me the week before. So we had an Italian lady, who wanted to do, like a tomato mozzarella pasta, warm. And so she discussed it with me on the Saturday, put it to all the women, would they like to try it the following week? So then this lady she come in, did it in front of everybody and then some of the women who wanted they could then try that at home. Do you know what I mean? So that's how some weeks we work it. Some weeks then, we'll say can we all have a go at that? You know, if it's like it's a unanimous decision, you're like, oh, yeah, I'd like to try making that then that's what we do. So there’s like little changes incorporated, but we make sure that everyone gets what they want.” (LGC007)*

*“So there's lots of different things we do when we also have, like a shared ideas. So some of the ideas will come from Let’s Get Cooking. But then some parents will say, well, we tweaked that recipe and we found by doing this, it was better for us or…So they've started taking a lot of the info, you know, on the batch cooking and doing a bigger amount and freezing it. And so we've purchased tubs for people to freeze things and and given the information you have lots of information about how to store things and how to freeze things and how to make sure that your defrosting them properly. So there's a lot of that kind of information that's gone out to parents as well as just the physical food and recipes.” (LGC004)*

***3.3.2. Perceptions of training***

*“I've definitely learned something really useful around, you know, how to cut an onion properly or pineapple and stuff with children, you know, all the sort of stuff that you learn that whenever you're an adult cooking for yourself, you just do it, don't you? But whenever you've got children with sharp knives, you have to kind of know what you're doing” (LGC001)*

*“The the training we did receive was really good...It was good from the the point of view that we, you know, the skills that were discussed and, you know, knife skills and how to, you know, use different ways of cutting.” (LGC004)*

*“There was some nice little tricks that the training did where, you know, for the children especially, say them using the claw technique or the or the bridge technique. There were, there were little tips about putting a fork in an onion and then you can hold the handle of the fork, which is away from the knife and then slice them through the gaps of the fork. Those are the sorts of things that we will teach our children and also using scissors for cutting herbs and spices rather than having to chop them with a knife” (LGC005)*

*“I think the food hygiene was very good. It was very clear and it kind of was around, it was just that reminder to really to set up your routine for the clubs right from the beginning and just kind of reinforce it every single week so that you get to a stage after a few weeks whenever you're going into the practical room, they just straight away, you know, take the jumpers off, put their aprons on, go wash their hands, put the hair back, sort themselves out, and then if you get them involved in the cleaning as well at the end.” (LGC001)*

*“I learnt, how to read to be honest, I've never used a recipe book in my life. I'd normally like cook from scratch and not use anything like that. But she taught me how to, you know how to use a recipe book for a start, which was good for me.” (LGC003)*

*“It was, I think it was really good in that it helped us understand why the programme was set up and what its aims were and what the values of Let's get cooking are So and I think and then so that, so I found that really really useful because I think it adds real value to to the club…And then actually, there were some really helpful tips. That that just about just about the setting it up and the communicating with parents and little things, you know, just the risk assessments and things like that. So yeah, I it was, it was it wasn't too much. But I sort of, having started off thinking I don't really need to do it, I it was helpful.” (LGC006)*

*“…it's having that confidence and I think that that kind of has been something that's come from the Let’s Get Cooking is being the confidence to to go no this is going to work and to persevere with it.” (LGC004)*

*“Their overall plan and delivering of session as well, like made me really confident and comfortable” (LGC007)*

*“So I've done the training a few times and then we've had Let’s Get Cooking actually come into our school for a whole day in the past where they did training for about 15 members of members of staff in the school and that was all funded because one of the Let’s Get Cooking ladies, she supported us with an application form to get some of that kitchen equipment as well. So that was that was amazing.” (LGC005)*

*“So yeah, for me the the more practical, the better, because we’re already doing the stuff. There wasn't, there wasn't too much talking going on. So but really important to know, you know how to chop, how to peel, little little hints and tips on how to chop an onion and stuff like that was really good, which we could pass on back to our children” (LGC005)*

*“[Training] was online, it was online. It was a little clunky to start with. I think one of the trainers couldn't get on, so that was a little bit chaotic. But apart from that, once we got going, it was fine.” (LGC006)*

*“I prefer in person because then obviously your tutor’s right there, you know, so and anything you’re unsure of straight away. And plus, you're leaning from others as well, you know, around you and stuff. So yeah, I prefer definitely in person, 100%.” (LGC007)*

*“So the training for this year was just very sort of an online training session. I would say that if you were new to delivering Let’s Get Cooking or any cookery sessions I would say it was very limited if I'm absolutely honest, I think you would possibly struggle to run a cookery club really well. The in previous years when Let’s Get Cooking started out, it was a proper sort of programme of training that was delivered to the people that volunteered to do it. So the training sessions were sort of ran over so many weeks and you actually had to go to a centre and have practical training skills as well that was delivered to you. And then it was ongoing. So you would regularly have updates to keep the training in place. So obviously, as I've been doing it a long time under the old programme, I've had lots of Let’s Get Cooking training delivered to me. So when I um volunteered for this one and we got the £250 for this year. I was quite surprised that it was quite limited the training, it was just a couple of online sessions.” (LGC008)*

*“So I think that's something that perhaps should be in the start of the programme, actually teaching someone how to teach the children how to use knives safely. You know, the bridge method, different types, that sort of thing. That wasn't part of the the training programme. So though the training programme sort of told you about, Let’s Get Cooking and gave you some examples of how to run a club. I don't think the training actually gave the people doing it the skills to teach the children, if that makes sense.” (LGC008)*

*“it would have been more beneficial to have done it where the person like yourself is there delivering the training, perhaps cooking some of the recipes or going through some of the steps to show and especially things around knife skills, it would be good to have that taught online.” (LGC008)*

*“I think it's perfect to be honest with you. Yeah. Yeah, yeah. Because sometimes you do get a bit lax, I think even in my line of work, we always have to have consistent training just to keep it up there Do you know what I mean? So yeah, I well appreciate ongoing training.” (LGC007)*

*“Having training within a school where a lot of the staff can be a part of it rather than me just being part of it and bringing it to the school. Having like half a day or a day which was funded where you would come in and do loads of recipes with a load of members of staff. That way you've got a lot more people trained at once. So if we're doing after school clubs, they're all they're all, Let’s Get Cooking trained and we could do a lot more cooking activities…So that would be a big benefit…the more people trained in one environment for my purp, for my school in particular, is a real benefit and it's happened once before. So that would be my main benefit of how to of how to get more staff involved within in a school” (LGC005)*

*“Have [training] more available because I say I haven't been, I've been to any training I'd say for about, gosh, it could be about 6 years. It could be even longer, you know. To be able to maybe have a main training session and then like even if it was an online like refresher session or you know a skills sharing session or a recipe session or just something you know to maybe annually or biannually” (LGC004)*

*“Yeah, the the coverage was more than enough, I think for people who have done the training before, if you do the training again, a lot of it is quite repeated, so the coverage is is more than enough. And you know it's a bit like children really, the quicker we you get into the actual practical stuff, the more exciting and fun it is and then we can learn from our mistakes you know a lot quicker.” (LGC005)*

*“I think the training's fine. The training is actually really good. It's very beneficial. A lot of it, if you've already done the training once, you don't need all of it so. I know, it's a it's a case of resourcing and it's a case of whether you've done the training before. So if I've done the training before, I don't need a lengthy spiel on how to cut and how to chop and how to peel but what I do need is being taught how to cook hot food and then being given the resource to take back to school” (LGC005)*

***3.3.3. Adapting for participant needs***

*“In terms of the club itself, it has been run as an after-school club in the past, but we've moved away from that because we want to. My role, I'm I'm known as an achievement advocate, which you don't really get in primary schools, so a lot of my role is to do with bringing achievement into school in different ways. So one of that is health, fitness, getting parents involved into school and I also coach a lot of children back into class. So when they're struggling in class, we meet together and stuff. So we've been incorporating Let’s Get Cooking into those children. So sometimes there’s a group of four or five girls, sometimes there's a group of boys, sometimes a mixed group. And sometimes it's just like. Some of our really vulnerable children who just need time out of class, so we access Let’s Get Cooking with them as part of our healthy schools programme as well, so we we integrate it into that. We also do food technology in the higher year groups. So some of the class teachers they use the Let’s Get Cooking programme within their classroom. So thirty children at a time are being accessed with that programme, so it's all sort of intermingled and merged with a few different things dotted around the school” (LGC005)*

*“…previously we've done [cooking clubs] as lunchtime. We've done them as after school curriculum as well. Yeah, it depends what we can fit in with within our school” (LGC008)*

*“We try and make it short and sweet and everybody's happy. They don't like to be sat down for long periods of time. Our kids, you see, they like to be on the go. So we tried to do a short session and move on to something else.” (LGC003)*

*“You know it's children with, you know, difficulties with fine motor skills, children with sensory issues, you know who don't like putting their hands in things. Sort of. You know, we've got one child who's got dyspraxia, so sort of there's a lot of mess everywhere. But but it's not. It doesn't really come down to age and you know, you just, you know, that's the same as being in class with these children. You sort of just have to accommodate and adapt for their needs” (LGC006)*

*“We've got a mixed population in school, mostly British White, but we have got lots of different cultures and backgrounds within our school. So of I mean, we do vegetarian cooking anyway, but we would have to do that because obviously a lot of our students would have to hal halal meat which I can't say. So yeah, so we've got a a mixed bag of of children within school. Children from all sorts of backgrounds. Children looked after children. Children new to the country, children where English isn't their first language. So yeah, so so we have a lot of different types of children with issues that we have to accommodate” (LGC008)*

*“The the year four thing would be probably a slightly simpler, slower recipe, yeah. And. Probably slightly more clearly outlined exactly what we're doing and much more kind of reading from the through the recipe at the beginning, making sure that everybody knows what's going to be happening.” (LGC001)*

*“It's more of a structured session. They’re both structured sessions, but with the juniors, obviously we'll have to do a lot more for them. So we'll give them the mixing bowls and the this, that and the other and we'll give them all the ingredients and we'll help them do the maths with the weighing and this, that and the other way as the seniors can pretty much do things for themselves if that makes sense” (LGC003)*

*“OK, so obviously I have to, its age appropriate and who I've got. We, I tend to always sit with them to find out what it is they'd like to cook. They'll come up with wild, wild and wacky suggestions and then sort of pare it back. I try, depending on their age” (LGC008)*

*“It's open for year one to year six, so our youngest will be 5 and our oldest will be 11 and it's a complete mix of all age groups and the sort of rationale behind that was one, because we wanted siblings because you can't, a parent’s not going to come at 3:00 o'clock to pick one child up, go away for an hour and come back to pick the other child up. So that would sort of prevent some children from being able to attend. And also we we sort of let the older children. So we when we sit to them on tables to do the work, we’ll put so so many younger children with so many older children, so the older children can help the younger children.” (LGC004)*

*“I have to just deliver a slightly different, a lot of our children who we have in this centre, I'd say over 90% of them have got additional needs or behavioural issues. So it's just about working slightly different with different groups, if that makes sense…Some might need a little bit of extra help, others might not. It's just so yeah, the training obviously works and helped, but I've just got to deliver it slightly different.” (LGC003)*

*“It's probably the same [resource] being used in a slightly different way. Yeah, or. And often by year six, you kind of know more about the food that they really like to make or the food that they really, you know…whereas kind of the year fours probably think they're going to turn into they don't know what.I mean, it's it's interesting too, because some of them they do like fruit and veg, they do like fruit and veg. They do. They definitely do like fruit and veg by year 6, whereas some of the year fours are just like I don't eat tomatoes. I don't eat this. I don't eat that, which is where the old kind of you got to try it and then say you don't like it, which is fine. But no, similar resources, similar resources” (LGC001)*

*“Yes, it has because there's some things that I would, you know, sometimes you do something and it turns out to be a complete disaster. And I don't go back and do that again… but I think it does get slightly easier once you know the recipes that really work for you and the children” (LGC001)*

*“So I mean I'm working my way through the through the recipes that they gave us and adding a couple in. As I say, it sort of depends, it is very dependent on how much support I've got, what space we're going to be in.” (LGC006)*

*“I pretty much do what I was taught there. We do obviously do our own sessions as well in between. But these out of recipe books again, do you know what I mean? So it's all pretty much the same thing” (LGC003)*

*“…we've become a bit more diverse in Kirkby where I live. Kirkby was always historically a white British town where we've got lots of different. Huge selection, really. We've got a lot of Lithuanians. We've got quite a few people from Brazil. And we've got a few Slovakians now, a couple of people from Ukraine, so. It's been like. A personal challenge and and a challenge of them to make sure that you know they feel included. Being respectful of other people's cultures and maybe a little bit of ignorance on our behalf of certain food groups that certain cultures won’t eat….So maybe we need to be a bit more inventive and maybe a little bit more, sort of open minded on what goes in our recipes and the kinds of recipes we we do because we tend to stick to tried and tested” (LGC004)*

*“Maybe even have just a purely vegetarian stroke vegan recipe book or rec, or you know, structures ideas to supplement everything else…Maybe just consider having maybe a multinational or, you know, a multinational cookbook, as a supplement that we can dip into and like a vegan or vegetarian, or that we can dip into.” (LGC004)*

***3.3.4. Resourcing clubs***

*“I mean, the only problem is in the school, things disappear if you put things into the practical room and you think you've got so many bowls and then next week you haven't because some of them disappeared around school somewhere and then at the end of the year, somebody brings mouldy bowls back or whatever. But it's not. It's we've been well resourced. We have been well resourced, yeah” (LGC001)*

*“We were really well supported with with they sent us, we we sent a shopping list of equipment that we needed and that was that's really fantastic because it's the sort of thing that sort of, especially in little schools, you know, to go off and spend £120.00 on cookery equipment, sort of it's not always that easy to justify. So that was. Yeah, that's really valuable and it's really nice to be able to just that also makes it run really smoothly. I know I've got a knife between the two, a bowl between two, you know enough enough scales, things like that. It's nice to be well resourced. Actually, I think I think actually is more efficient actually just. Because I've got all those resources there.” (LGC005)*

*“We got a voucher off yourselves when we signed up last time. So we got loads of scales, loads of like, plastic bowls, wooden spoons, different equipment” (LGC003)*

*“Really, really, really use really useful, some really good recipes and really good at kind of timings and stuff, because actually you've got to get through quite a lot to produce something with 15 children and be washed up by the end of an hour and a quarter. Yeah. So yeah, it's it the recipes have been helpful” (LGC001)*

*“If we didn't get the funding, then you know, like I said, we're a Tower Hamlets based school, you know, the the squeeze on school budgets at the moment, I don't think Let’s Get Cooking would be one of those things that will continue. So. That small amount of money, which feeds into the school really helps. Where because every single budget and every single subject is stretched so much, when you go to the head teacher and say, oh, is there any chance we can get some money for buying food? It's like, OK, we'll have to think about it. And the answer generally is no” (LGC005)*

*“[Being self-sufficient] will never happen. Do, we just haven't got the money. You know, in an ideal world, in a fantastic affluent area then yeah, you know, one of the staff here, her brother lives in Northampton and he pays £5 a week for a cooking session. And yeah, she goes, they don't even make a lot. You know what they're doing, they don't even bring home what they do and I'm like. Yeah. You can't. You can't. You can have all your ideals, and if you're in an area that's affluent, that's fantastic…but the clubs will never be self-sufficient. It just can't happen” (LGC004)*

*“I think that obviously if [funding stops] at some stage and then the money would have to be found from somewhere else or we'd have to charge for the clubs or we'd have to, it's kind of it's it's a difficult one and the ingredients are quite it's. You'd have to work out roughly the actual cost of what it would be costing you to run it, wouldn't you, which you know. It's it's a, it's a. It's tricky one. It's a tricky one.” (LGC001)*

*“It would be good to be self-sufficient yeah, but I'm not sure how that would actually happen, if that makes sense. Because at the end of the day to run a cookery, I mean basically if you run a cookery club you need ingredients that that if you've got the equipment it's just ingredients. So it's how are you going to fund that. So either you're going to have to charge the families to take part in it, which in where our school is, we're quite a deprived area, so I don't think that that would actually work if I'm absolutely honest. So therefore, we'd have to look out for other organisations to support us with our funding, yeah.” (LGC008)*

*“Well, what what it is with us is obviously, we're a charity, so we only get so much funding. So this is why I can't do the session every week. We try and tend to do the session a couple of times a month as opposed to every week” (LGC003)*

*“We see everything towards was cost absolutely everything to us is cost.So we did used to ask the children for a nominal fee just to cover the ingredients, but if they didn't pay, that didn't stop them coming, you know” (LGC004)*

*“We're running it ad hoc at the moment. So it isn't because just because again, it's sort of my capacity…So it's not so it isn't. It isn't run every week, but it's all. It's all diaried so the parents know when it's coming up…So we had to fit it around other commitments.” (LGC006)*

*“I suppose there could be probably shorter recipes. A few more shorter recipes, maybe a little bit more kid friendly. So that obviously they'll enjoy it a little bit more if that makes sense because some of them are a little bit long. And they involve a lot of ingredients, so obviously that takes longer to do, but it works well. It's fine. It's just we only have two hours” (LGC003)*

*“I suppose it depends on the equipment really what we use because one of the biggest barriers we always have is having the right equipment to cook with. So you know, for example, those the stoves that, that you can get the induction stoves, they showed us how to cook a really healthy pizza. Well, we haven't got a conduction stoves, you know, in our school. We’re a Tower Hamlets based school and we just haven't got the money. So you know those, those aspects of of the training are great, but it doesn't really help us because we're never going to get an induction stove. So those are the sorts of things that are not really helpful” (LGC005)*

*“But in previous years when we've done, Let’s Get Cooking clubs, we've had a bigger number of children. There's been two members of staff. There's been, myself and someone else who's led it with me. But that member of staff has left now. So obviously there isn't anybody who can do it with me. So that's why it's been a smaller, smaller groups this this time round. Yeah.” (LGC008)*

*“Yeah, just shorten the session down a little bit. So obviously, for example, if we're making the pizzas, I've got all the ingredients on the table. It's all there ready for them to do. The session will probably be about 15-20 minutes. Be prepared. They'll make them and then we will cook them. So we'll obviously in the recipe books you cook it yourself. But we'll do that side for them. So we just shorten it down, that's all.” (LGC003)*

*“I don't know whether I should say this, but literally 'cause I feel passionately about children cooking, I pay for all the ingredients that get used out of my own money. Because I want the cookery club to continue. I want the children to have that experience and I want them to, you know, be able to cook something and take it home and be proud of what they've made and hope that they do learn a skill and that, it will be something that will stay with them” (LGC004)*

*“I think with with a with a whole class environment, it's a little bit more difficult for everybody to you know, have an apron and everyone to have utensils, so it's very much done at the front where we show them how things work and then they get their chance to do the chopping you know, one between 4 or something like within a classroom. So the bigger the class the less chance they get to experience, all the chopping “ (LGC005)*

*“What could be maybe included is in the recipe books, it maybe could tell you, how much each recipe is going to cost, if that makes sense. So when you come to look at your recipes and you're seeing the price at the bottom or the top, you'd know, oh I couldn’t afford that this week because that's a little bit dearer” (LGC003)*

*“I mean, this is quite blunt, but it just boils down to the fact that if we're gonna, if we're gonna get training on cooking particular types of recipes and healthy recipes for the children, and they require certain equipment, then that's what equipment needs to be given to the schools in order to use them.” (LGC005)*

*“I think possibly if you were starting out, though obviously we you get so much money, I don't think that would be enough to actually set you up with a cookery club. So it might be nice, but obviously I know it comes down to funding, that they actually gave each group perhaps a basic cookery pack with the basics in so that they've got that to start up a cookery club. So that would be like mixing bowls, wooden spoons, scales that type of thing, so they've actually got sort of a pack of what you can get from a cookery group. Yeah. Or even send a list, or even send like a list through of like, you know, a very basic cookery group, you need this equipment to to start it. There is, there really wasn't much guidance around that at all.” (LGC008)*

*“Because often in recipes you know, what was a Jamie Oliver's 15 minute meals or something that took everybody 40 minutes. Yeah. So the Let’s Get Cooking stuff does kind of do what it says on the tin. If it says this is going to take you, you know, 30 minutes and 10 minutes to it it works” (LGC001)*

*“Yeah, we've, we've had financial support and then we had some resources as I was saying to you before, we had like these, you know, plastic measuring cups and some jugs and things. And we used to get equipment used to get a catalogue where you could choose equipment, many years ago. And then that sort of I think that's petered out, I think they lost…I think that all that funding went” (LGC004)*

*“It's difficult because as I say, like historically with the Let’s Get Cooking website, there was lots of information on there, lots of recipes. It's, the BDA have got the website, and they have got some Let’s Get Cooking on there but it's not quite the same as what it was historically. And I mean like they used to supply certificates and things that you could give out to the children. Obviously, that doesn't happen in quite the same way. It is. It is a, it is. It is a different scheme to how it used to be, but it's just under the banner of Let’s Get Cooking, yeah.” (LGC008)*

*“The other thing that historically Let’s Get Cooking would do was they provided you with lots of booklets and training guides, which obviously you don't get anymore. I mean, I've still got mine because I kept keep everything. So I did reach it out. I don't know if you’d like to see it. So there was a whole, there was a whole training programme that you had to work your way through. Let’s Get Cooking would issue you with recipe books as well, so you'd have like a winter one, seasonal cookery. So like every term there would be a cookery book produced for you to work through if you wanted to. Obviously that hasn't happened this time round and I don't think it's something that the BDA do with the Let’s Get Cooking group.” (LGC008)*

***3.3.5. Networking***

*“[Training] was also good 'cause there was other people from other schools and they were saying this works good in our school, this doesn't work so well in our school. We've tried this and we've tried that. And you know, because you don't want to, you don't want to feel like you're the only one sitting there who's like, well, that just wouldn’t work in our school…talking with other people was really useful, you know, really good. And it was you tried doing this and we've done that and things you know” (LGC004)*

*“I was encouraged a lot and I was supported a lot. It has fragmented, partly probably down to us, partly down to the actual structure of Let’s Get Cooking, I'm not sure. But I think in the early years when I was sort of out there on my own, trying to establish a cooking club, you know, it was great to have that support structure there. And to to have the opportunity to meet, to net, to network with other schools and all the people who are doing the same” (LGC004)*

*“I think [community or support] probably reduced a bit in the last year or so. Yeah, that's probably reduced…I think I think it looked it used to be sort of there'd be a session or something that you'd go to for training or. And you would meet other people from other schools that were doing the same thing. And and that hasn't happened since COVID. And I think that's probably I understand everybody's really, really busy and everybody’s sort of doing stuff, but it is quite nice just to hear what other people are doing actually…'cause there used to be a newslettery thing years ago, but kind of that’s thrown up the bell, but there used to be a newsletter thing that sort of said various things that various schools were doing and stuff, which was always worth reading. Yeah, but nothing. Yeah, but I think that's sort of across the board, really, the sort of a lot of those old links are sort of diminished slightly.” (LGC001)*

*“[Training] was good from a networking point of view because you could talk to other people and say, you know we've got this problem with our cookery group, a lot of networking, a lot of input and a way of solving problems for your cookery group” (LGC008)*

*“So after the initial, obviously because it was area based, you would know like where your next local school was that was running cookery group. So you could connect with them. Even just by emails or just having a chat to sort of say you know we've run into this issue, it was just somebody out there that you could chat through things, especially when you start involving children with special educational needs into your groups. Sometimes it was good to be able to talk to other schools that were doing that to get some ideas of how you could, you know, adapt things to make it more productive for those children.” (LGC008)*
